# Amelioration of Pulmonary Fibrosis by Matrix Metalloproteinase-2 Overexpression

**DOI:** 10.3390/ijms24076695

**Published:** 2023-04-03

**Authors:** Ryo Inoue, Taro Yasuma, Valeria Fridman D’Alessandro, Masaaki Toda, Toshiyuki Ito, Atsushi Tomaru, Corina N. D’Alessandro-Gabazza, Tatsuki Tsuruga, Tomohito Okano, Atsuro Takeshita, Kota Nishihama, Hajime Fujimoto, Tetsu Kobayashi, Esteban C. Gabazza

**Affiliations:** 1 Department of Immunology, Mie University Graduate School of Medicine, Tsu 514-8507, Japan; 2 Central Institute for Experimental Animals, Kawasaki 210-0821, Japan; 3 Department of Diabetes, Metabolism and Endocrinology, Mie University Graduate School of Medicine, Tsu 514-8507, Japan; 4 Department of Pulmonary and Critical Care Medicine, Mie University Graduate School of Medicine, Tsu 514-8507, Japan

**Keywords:** matrix metalloproteinases, pulmonary fibrosis, matrix degradation, apoptosis, collagen deposition

## Abstract

Idiopathic pulmonary fibrosis is a progressive and fatal disease with a poor prognosis. Matrix metalloproteinase-2 is involved in the pathogenesis of organ fibrosis. The role of matrix metalloproteinase-2 in lung fibrosis is unclear. This study evaluated whether overexpression of matrix metalloproteinase-2 affects the development of pulmonary fibrosis. Lung fibrosis was induced by bleomycin in wild-type mice and transgenic mice overexpressing human matrix metalloproteinase-2. Mice expressing human matrix metalloproteinase-2 showed significantly decreased infiltration of inflammatory cells and inflammatory and fibrotic cytokines in the lungs compared to wild-type mice after induction of lung injury and fibrosis with bleomycin. The computed tomography score, Ashcroft score of fibrosis, and lung collagen deposition were significantly reduced in human matrix metalloproteinase transgenic mice compared to wild-type mice. The expression of anti-apoptotic genes was significantly increased, while caspase-3 activity was significantly reduced in the lungs of matrix metalloproteinase-2 transgenic mice compared to wild-type mice. Active matrix metalloproteinase-2 significantly decreased bleomycin-induced apoptosis in alveolar epithelial cells. Matrix metalloproteinase-2 appears to protect against pulmonary fibrosis by inhibiting apoptosis of lung epithelial cells.

## 1. Introduction

Pulmonary fibrosis is the most advanced stage of a group of lower respiratory tract inflammatory disorders categorized as interstitial lung diseases (ILD) [1]. Idiopathic interstitial pneumonias are the main subgroup of ILD, representing about 40%, and of these, a subgroup classified as idiopathic pulmonary fibrosis (IPF) is the most common form, accounting for more than half of idiopathic interstitial pneumonias [2]. Age-adjusted incidences of 16.3 per 100,000 per year for broad criteria, 6.8 per 100,000 per year for narrow criteria, and 93.7 per 100,000 per year in a population aged 65 or older were reported in the United States of America [3,4]. Recent worldwide epidemiological data suggest that disease prevalence is rising [5]. There are approximately five million IPF patients worldwide. There is no apparent difference in incidence or prevalence among ethnic groups, but the disease is more frequent in males and generally associated with a worse prognosis [5]. IPF is a progressive and fatal disease with a median survival of only 2 to 4 years following diagnosis and 5-year survival of only 20%. Thus, it is considered more lethal than many types of cancer [6]. Genetic and environmental factors contribute to the risk of developing IPF [7]. However, no definite etiology of IPF has been identified, and no effective therapy currently shows survival benefits [1]. The increased prevalence and poor prognosis of IPF urge the development of effective therapeutic strategies.

The initial insult that causes IPF is still unknown, but once the disease is triggered, fibroblasts producing excessive extracellular matrix (ECM) proteins (e.g., collagen, tenascins, fibronectin, periostin) accumulate in the lungs [1]. Lung scarring may result from a disturbance in the balance between the proliferation and death of fibroblasts or between the production and degradation of ECM proteins. Pulmonary fibrosis ensues when the balance shifts toward fibroblast accumulation and increased ECM protein deposition. Fibroblasts may be resident lung fibroblasts and/or originate from fibroblastic differentiation of bone marrow-derived cells (circulating fibrocytes or monocytes) or epithelial–mesenchymal transition. Activation of signal pathways resulting from the cross-talk between metaplastic alveolar epithelial cells and fibroblastic foci is also an important contributor to the pathogenesis of lung fibrosis [8]. In addition, growth factors (e.g., transforming growth factor [TGF]β1, connective tissue growth factor, platelet-derived growth factor), inflammatory cytokines (e.g., interleukin [IL]-13, tumor necrosis factor α [TNFα], IL-1β), chemokines (e.g., monocyte-chemoattractant protein-1 [MCP-1]), and clotting factors (e.g., thrombin) contribute to migration, recruitment, and activation of fibroblasts during pulmonary fibrosis [9,10,11,12]. On the other hand, the cleavage of ECM proteins is regulated by the proteolytic activity of matrix metalloproteinases (MMPs) [11]. Many MMPs have been identified so far [11]. Several studies reported enhanced activity of MMPs in idiopathic pulmonary fibrosis [13,14,15,16,17,18]. However, discrepant effects of MMPs in the process of pulmonary fibrosis have been reported [19,20,21] (Supplementary Table S1). MMP-3, -8, -11, and -28 are profibrotic, while MMP-10 and -19 are antifibrotic [22,23,24,25,26,27,28,29]. MMP-1, -7, -13, and -14 have been reported to promote or suppress fibrosis [30,31,32,33,34,35,36,37,38,39,40]. Profibrotic activity, antifibrotic activity or no effect on collagen deposition has been reported for MMP-9 and MMP-12 [21,41,42,43,44,45,46,47,48,49]. The roles of MMP-15, -16, -17, -20, -21, -22, -23, -24, -25, -26, and -27 in lung fibrosis remain unexplored [19].

MMP-2 expression is also increased in patients with ILD, suggesting its potential implication in the pathogenesis of pulmonary fibrosis [14,15,16]. However, there are contradicting reports on the role of MMP-2 in the fibrotic process. Previous studies have attributed either a profibrotic activity or no effect on fibrosis to MMP-2 [42,50]. The present study aimed to evaluate the effect of human MMP-2 overexpression on the development of pulmonary fibrosis in a bleomycin mouse model.

## 2. Results

### 2.1. No Difference in Body Weight between WT and hMMP-2 TG Mice with Lung Fibrosis

Mice were administered BLM or saline through osmotic minipumps for seven days, and the development of fibrosis was evaluated on day 21 after the first day of BLM infusion (Supplementary Figure S1A). The body weight of the mice was measured daily. The body weight was decreased in mice receiving BLM (WT/BLM, hMMP-2 TG/BLM) compared to mice receiving saline (WT/SAL, hMMP-2 TG/SAL). However, there was no significant difference in body weight between WT/BLM and hMMP-2 TG/BLM mice (Supplementary Figure S1B). In addition, no mortality was observed in mice receiving BLM or saline. These observations suggest that BLM was successfully delivered to each mouse of the WT/BLM and hMMP-2 TG/BLM groups.

### 2.2. Decreased Inflammatory Cells in the Lungs from hMMP-2 TG Mice

**Figure 1:**
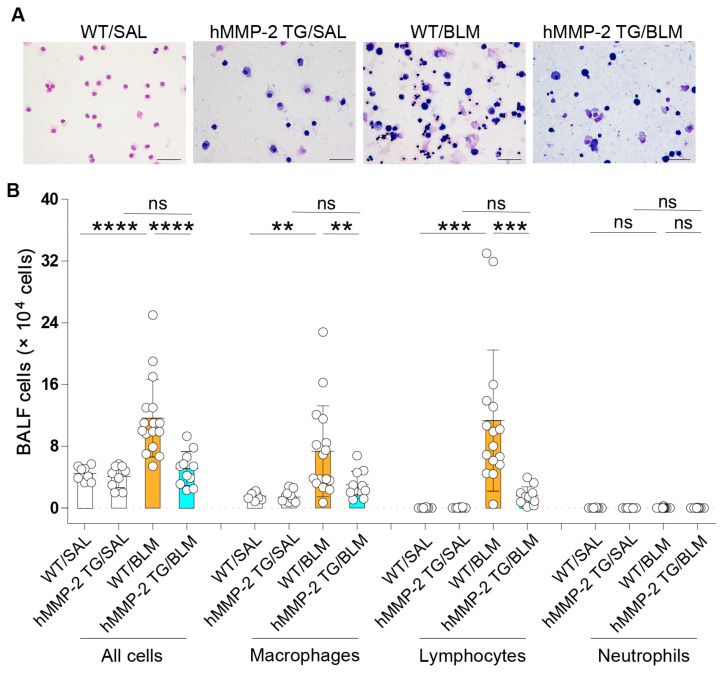
Decreased inflammatory cells in the lungs from hMMP-2 TG mice. The total number of cells in bronchoalveolar lavage fluid was measured with a nucleocounter (**A**). The cells were stained with Giemsa to perform differential cell count. Differential cell count is expressed as the total number of each cell type (**B**). The number of mice: n = 8 in WT/SAL, n = 9 in hMMP-2 TG/SAL, n = 16 in WT/BLM, and n = 11 in hMMP-2 TG/BLM. Scale bars indicate 20 µm. All mice were female. Data are the mean ± S.D. Statistical analysis by ANOVA with Newman-Keuls test. **** *p* < 0.0001; *** *p* < 0.0001; ** *p* < 0.0001. WT, wild-type; hMMP-2, human matrix metalloproteinase-2; SAL, saline; BLM, bleomycin; ns, not significant.

The total number of cells in BALF was determined using a nucleocounter, and a differential cell count was performed after Giemsa staining. The total number of all cells and the total number of macrophages and lymphocytes in BALF were significantly enhanced in WT mice receiving BLM through osmotic minipumps compared to WT mice receiving saline and hMMP-2 TG mice receiving BLM. However, no significant difference in the number of cell types was observed between hMMP-2 TG/SAL and hMMP-2 TG/BLM groups or between WT/SAL and hMMP-2 TG/SAL groups (Figure 1A,B). These observations suggest the protective activity of MMP-2 overexpression against infiltration of inflammatory cells in BLM-induced lung fibrosis.

### 2.3. Reduced BALF Levels of Inflammatory Cytokines and Total Protein in hMMP-2 TG Mice

**Figure 2:**
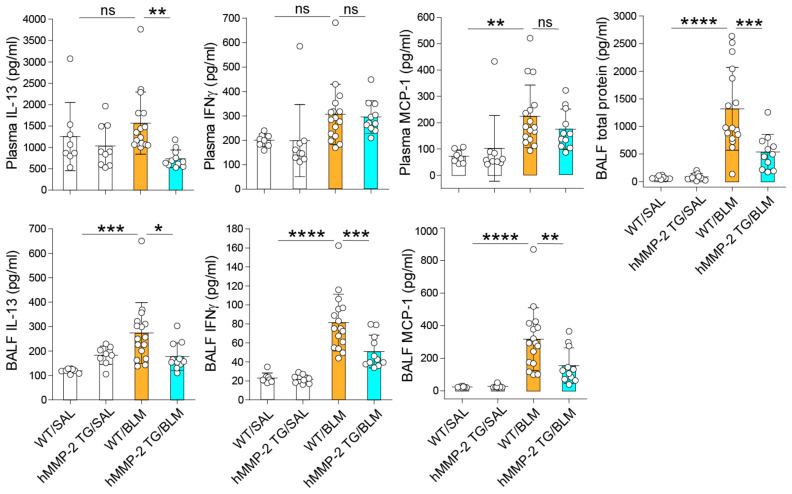
Reduced inflammatory cytokines and total protein in hMMP-2 TG mice. Inflammatory cytokines and chemokines were measured by commercially available enzyme immunoassay kits following the manufacturer’s instructions. The number of mice: n = 8 in WT/SAL, n = 9 in hMMP-2 TG/SAL, n = 16 in WT/BLM, and n = 11 in hMMP-2 TG/BLM. Data are the mean ± S.D. Statistical analysis by ANOVA with Newman-Keuls test. * *p* < 0.05; ** *p* < 0.01; *** *p* < 0.001; **** *p* < 0.0001; WT, wild-type; hMMP-2, human matrix metalloproteinase-2; SAL, saline; BLM, bleomycin; ns, not significant.

**Figure 3:**
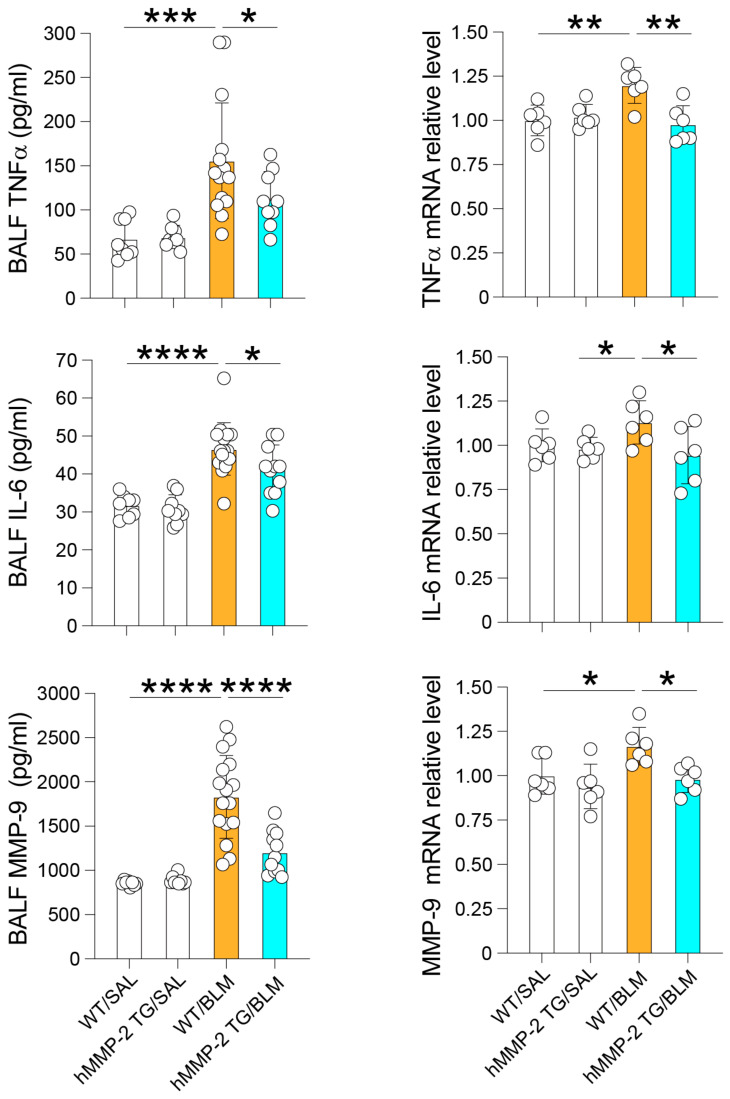
Reduced BALF and relative mRNA levels of TNFα, IL-6, and MMP-9 in hMMP-2 TG mice. Inflammatory cytokines and chemokines were measured by commercially available enzyme immunoassay kits following the manufacturer’s instructions. The number of mice: n = 8 in WT/SAL, n = 9 in hMMP-2 TG/SAL, n = 16 in WT/BLM, and n = 11 in hMMP-2 TG/BLM. Data are the mean ± S.D. Statistical analysis by ANOVA with Newman-Keuls test. * *p* < 0.05; ** *p* < 0.01; *** *p* < 0.001; **** *p* < 0.0001. BALF, bronchoalveolar lavage fluid; TNFα, tumor necrosis factor α; WT, wild-type; hMMP-2, human matrix metalloproteinase-2; SAL, saline; BLM, bleomycin.

Inflammatory cytokines were measured by immunoassays and compared between the different mouse groups. The plasma level of IL-13, but not that of IFN-γ or MCP-1, was significantly decreased in the hMMP-2 TG/BLM group compared to the WT/BLM group. The BALF concentrations of IL-13, IFN-γ, and MCP-1 were significantly lower in the hMMP-2 TG/BLM and WT/SAL groups than in the WT/BLM group (Figure 2). The BALF concentration of total protein, a marker of increased vascular permeability and lung inflammation, was significantly reduced in hMMP-2 TG/BLM mice compared to WT/SAL and WT/BLM mice (Figure 2). In addition, the BALF concentrations of TNFα, IL-6, and MMP-9 were significantly reduced in the hMMP-2 TG/BLM group compared to the WT/BLM and control groups (Figure 3). The decreased number of inflammatory cells and decreased levels of total protein and inflammatory cytokines suggest that MMP-2 overexpression protects against inflammation in BLM-induced lung fibrosis.

The protein concentrations of TGFβ1 in plasma, BALF, and lung tissue, as measured by enzyme immunoassay, were not significantly different between WT/BLM and hMMP-2/BLM groups (Supplementary Figure S2A–C). However, the relative expression of TGFβ1 mRNA was significantly decreased in the hMMP-2 TG/BLM group compared to the WT/BLM group (Supplementary Figure S2D). Western blotting showed no significant difference in the protein expression of TGFβ1 between WT/BLM and hMMP-2/BLM groups (Supplementary Figure S2E,F). The different time course of TGFβ1 expression during the process of BLM-induced lung fibrosis may explain the discrepancy between the protein and mRNA expression of TGFβ1. Nevertheless, these results provide insufficient evidence to draw any conclusion about the effect of hMMP-2 on TGFβ1 expression.

### 2.4. The Levels of the MMP-2 Substrates IL-1β, Osteopontin, MCP-3, and CTGF Are Decreased in the Lungs of hMMP-2 TG Mice

**Figure 4:**
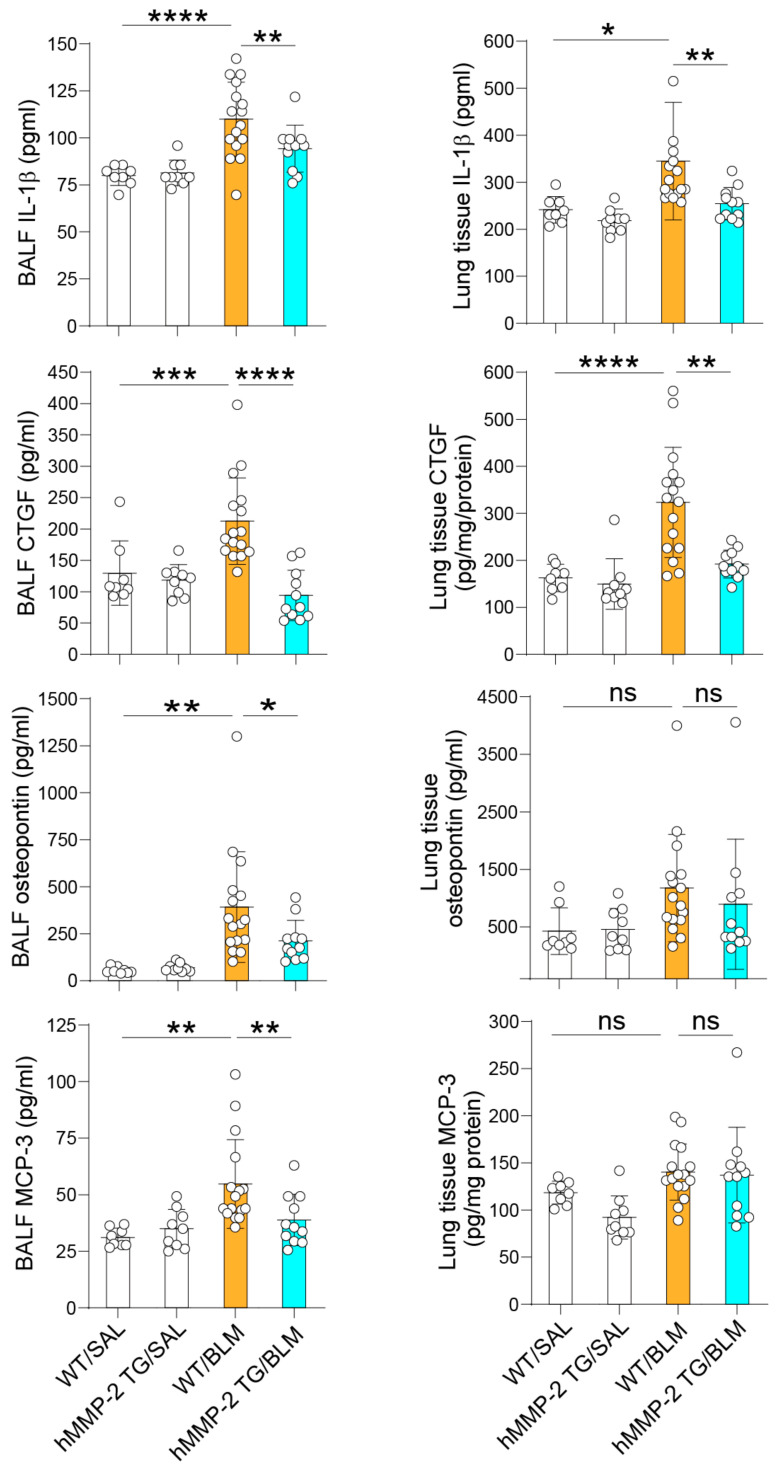
Substrates of hMMP-2 are reduced in the lungs of hMMP-2 TG mice. IL-1β, osteopontin, MCP-3, and CTGF were measured by commercially available enzyme immunoassay kits following the manufacturer’s instructions. The number of mice: n = 8 in WT/SAL, n = 9 in hMMP-2 TG/SAL, n = 16 in WT/BLM, and n = 11 in hMMP-2 TG/BLM. Data are the mean ± S.D. All mice were female. Statistical analysis by ANOVA with Newman-Keuls test. **** *p* < 0.0001; *** *p* < 0.001; ** *p* < 0.01; * *p* < 0.05. WT, wild-type; hMMP-2, human matrix metalloproteinase-2; SAL, saline; BLM, bleomycin, MCP-3, monocyte chemoattractant protein-3; CTGF, connective tissue growth factor; ns, not significant.

The inflammatory cytokines IL-1β and osteopontin, the inflammatory chemokine MCP-3, and the growth factor CTGF are well-recognized substrates of MMP-2 [51,52,53,54]. We compared the levels of these pro-inflammatory and pro-fibrotic factors in BALF and lung tissue. The BALF levels of IL-1β, osteopontin, MCP-3, and CTGF were significantly increased in the WT/BLM group compared to WT/SAL and hMMP-2-TG/BLM groups. The lung tissue levels of IL-1β and CTGF increased significantly in the WT/BLM group compared to WT/SAL and hMMP-2 TG/BLM groups. The lung tissue levels of osteopontin or MCP-3 were not significantly different between groups (Figure 4). In addition, the BALF level of platelet-derived growth factor (PDGF) was significantly increased in the WT/BLM group compared to WT/SAL and hMMP-2/BLM groups (Supplementary Figure S3). These results further support the anti-inflammatory activity of hMMP-2 in BLM-induced lung fibrosis.

### 2.5. mRNA Expression, Protein Level, and MMP-2 Activity in the Lung of hMMP-2 TG Mice

As expected, the mRNA expression of human MMP-2 was high in the lungs of both hMMP-2 TG/SAL and hMMP-2 TG/BLM mice. The mRNA expression of mouse MMP-2 was significantly higher in the WT/BLM and hMMP-2/SAL groups than in the WT/SAL group, but no difference was observed between WT/BLM and hMMP-2 TG/BLM groups (Supplementary Figure S4A). The protein level of MMP-2 in BALF was significantly increased in the hMMP-2 TG/SAL group compared to the WT/SAL group and in the hMMP-2 TG/BLM group compared to the WT/BLM group (Supplementary Figure S4B). It is worth noting that the immunoassay for human MMP-2 cross-reacts with mouse MMP-2. The level of MMP-2 by zymography was also significantly increased in hMMP-2 TG/BLM mice compared to WT/BLM mice and in hMMP-2 TG/SAL compared to WT/SAL mice (Supplementary Figure S4C,D). These findings suggest that overexpression of hMMP-2 mediates the protective activity against lung fibrosis.

### 2.6. Decreased Levels of Collagen, α-Smooth Musth Actin, and Fibronectin1 I in hMMP-2 TG Mice

The protein level of collagen in BALF and the relative mRNA expression of col1a1 were significantly decreased in the hMMP-2 TG/BLM mice compared to WT/BLM mice. In addition, the relative mRNA levels of α-smooth muscle actin, fibronectin, and tissue inhibitor of matrix metalloproteinase (TIMP-1) were significantly reduced in the hMMP-2 TG/BLM group compared to the WT/BLM group. The relative mRNA expression of vimentin was also reduced in hMMP-2 TG/BLM mice compared to WT/BLM mice. However, the difference was not statistically significant. No significant difference was observed in the relative mRNA expression of TIMP-2 and vimentin between WT/BLM and hMMP-2 TG/BLM groups (Supplementary Figure S5). Transforming growth factor (TGF)-α, a ligand for the epidermal growth factor receptor, has also been implicated in the pathogenesis of pulmonary fibrosis [55]. However, we found no difference in the expression of TGF-α between groups (Supplementary Figure S5). Overall, these observations suggest decreased expression of matrix components in mice overexpressing hMMP-2 with BLM-induced lung fibrosis.

### 2.7. Amelioration of Pulmonary Fibrosis in hMMP-2 TG Mice

**Figure 5:**
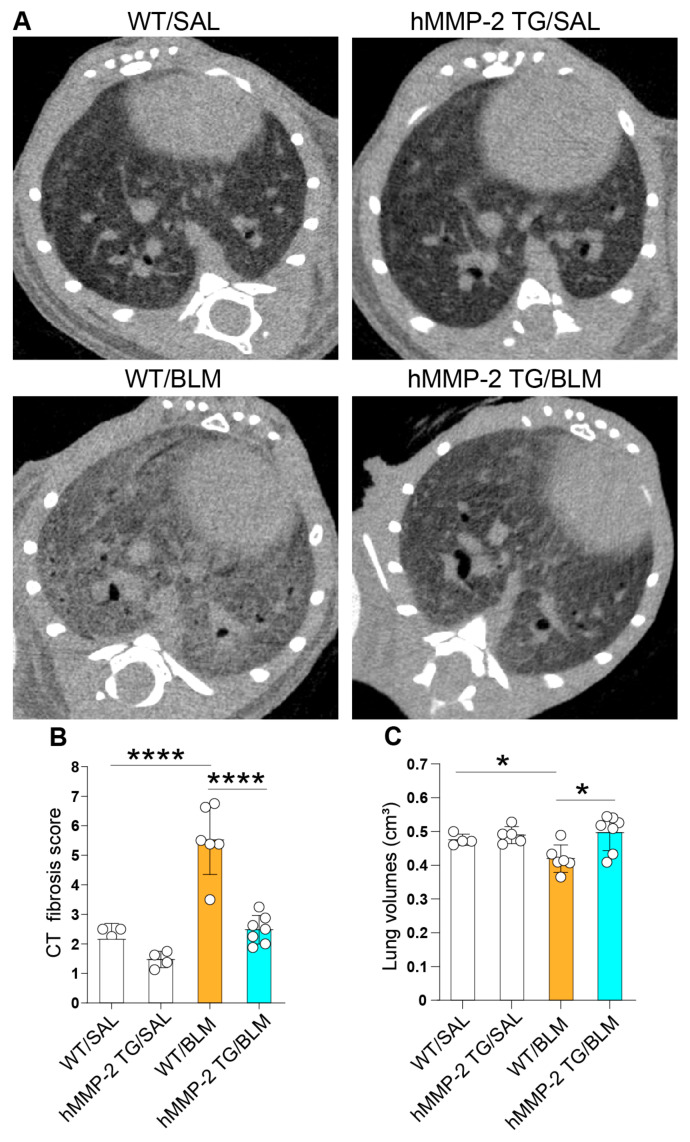
Amelioration of CT score of lung fibrosis and lung volumes in hMMP-2 TG mice. MicroCT was performed, and the radiological findings of fibrosis were scored by experts blinded to the treatment groups (**A**,**B**). Lung volumes were measured using microCT software (**C**). The number of mice: n = 4 in WT/SAL and hMMP-2 TG/SAL, n = 6 in WT/BLM, and n = 7 in hMMP-2 TG/BLM. Data are the mean ± S.D. All mice were female. Statistical analysis by ANOVA with Newman-Keuls test. **** *p* < 0.0001; * *p* < 0.05. CT computed tomography; WT, wild-type; hMMP-2, human matrix metalloproteinase-2; SAL, saline; BLM, bleomycin; TG, transgenic.

We performed microCT in mice from each group, and the radiological findings of fibrosis were scored by blinded experts for the treatment groups. The CT score was significantly lower in the WT/SAL and hMMP-2 TG/BLM groups than in the WT/BLM group. There was no significant difference in CT scores between hMMP-2 TG/SAL and hMMP-2 TG/BLM groups or between WT/SAL and hMMP-2 TG/SAL groups (Figure 5A,B). The lung volumes measured by CT were significantly decreased in the WT/BLM group compared to the WT/SAL and hMMP-2 TG/BLM groups. There was no significant difference in lung volume between hMMP-2 TG/SAL and hMMP-2 TG/BLM, and between WT/SAL and hMMP-2 TG/SAL groups (Figure 5C).

**Figure 6:**
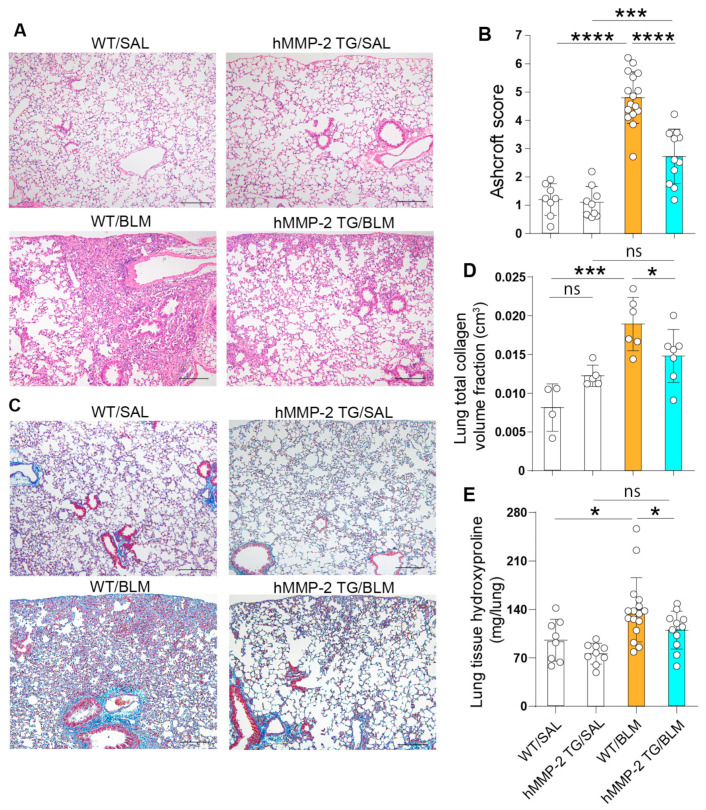
Amelioration of histopathological and biochemical findings of pulmonary fibrosis in hMMP-2 TG mice. Ashcroft scoring was performed in lung tissue stained with hematoxylin & eosin by blinded experts for the treatment groups (**A**,**B**). Collagen deposition was evaluated after trichrome staining, and the total lung collagen volume fraction was calculated (**C**,**D**). The lung tissue hydroxyproline content was measured by a colorimetric assay (**E**). Scale bars indicate 200 µm. The number of mice in (**A**–**C**,**E**): n = 8 in WT/SAL, n = 9 in hMMP-2 TG/SAL, n = 16 in WT/BLM, and n = 11 in hMMP-2 TG/BLM. Data are the mean ± S.D. The number of mice in D: n = 4 in WT/SAL, n = 5 in hMMP-2 TG/SAL, n = 6 in WT/BLM, and n = 7 in hMMP-2 TG/BLM. All mice were female. Statistical analysis by ANOVA with Newman-Keuls test. **** *p* < 0.001; *** *p* < 0.001; * *p* < 0.05. WT, wild-type; hMMP-2, human matrix metalloproteinase-2; SAL, saline; BLM, bleomycin; ns, not significant.

Evaluation of the grade of lung fibrosis was performed with the Ashcroft score using lung tissue stained with H&E. The Ashcroft score was significantly lower in the hMMP-2 TG/BLM group than in the WT/BLM group. The Ashcroft score differed significantly between WT/BLM and WT/SAL groups (Figure 6A,B). The total lung collagen volume fraction significantly increased in the WT/BLM group compared to the WT/SAL and hMMP-2 TG/BLM groups (Figure 6C,D). No significant difference was observed between hMMP-2 TG/SAL and hMMP-2 TG/BLM groups. The lung tissue hydroxyproline content was significantly increased in WT/BLM mice compared to WT/SAL and hMMP-2 TG/BLM mice. There was no difference between the hMMP-2 TG/SAL and hMMP-2 TG/BLM groups (Figure 6E). These observations suggest that hMMP-2 overexpression improves lung volume and protects against lung fibrosis.

### 2.8. Correlation between Lung Fibrosis and Inflammatory Markers

The relationship between markers of lung fibrosis and the number of inflammatory cells and the concentrations of cytokines and chemokines in BALF was evaluated in all groups. The markers of lung fibrosis, including CT score, Ashcroft score, total lung collagen volume fraction, and lung hydroxyproline, were significantly correlated with the number of macrophages and lymphocytes and the concentrations of cytokines and chemokines in all mouse groups. In addition, the BALF levels of hMMP-2 were significantly and inversely correlated with the total number of BALF cells and the BALF concentrations of CTGF and collagen (Supplementary Figure S6). These findings further support the role of MMP-2 in the process of lung fibrosis.

### 2.9. Inhibition of Lung Cell Apoptosis by hMMP-2 Overexpression

**Figure 7:**
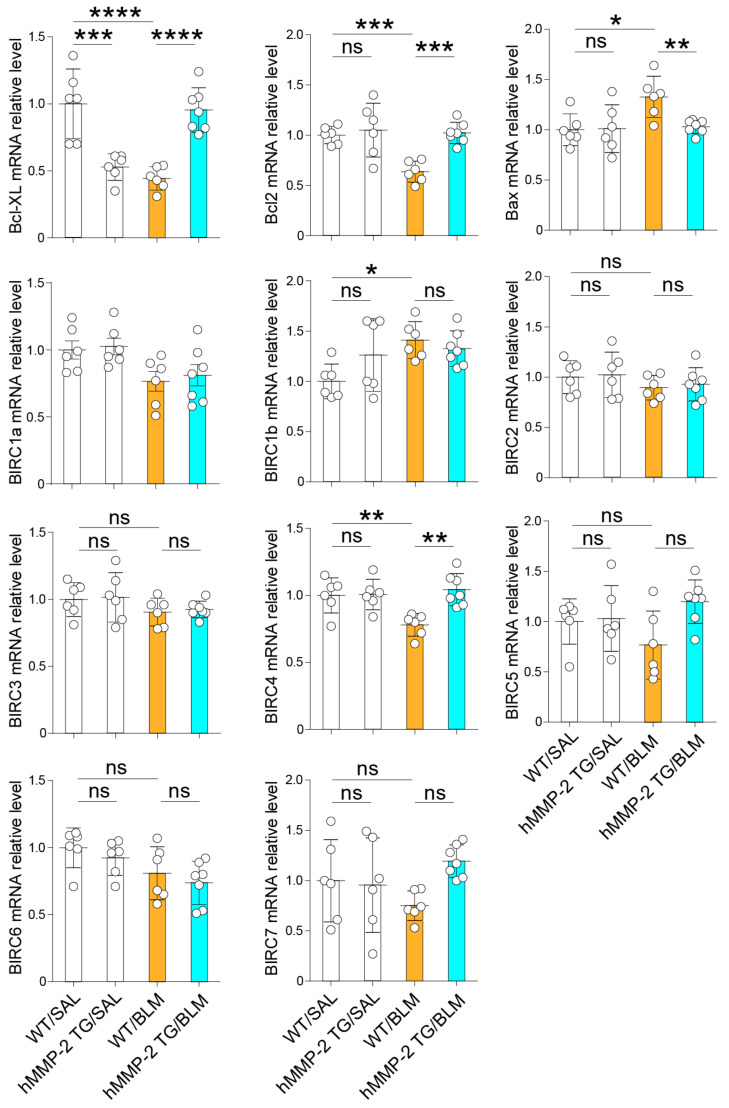
Inhibition of lung cell apoptosis by hMMP-2 overexpression. The total mRNA from the lungs of each mouse was extracted, and cDNA was synthesized using commercially available reagents. The number of mice: n = 6 in WT/SAL and hMMP-2 TG/SAL, n = 6 in WT/BLM, and n = 7 in hMMP-2 TG/BLM. Data are the mean ± S.D. All mice were female. Statistical analysis by ANOVA with Newman-Keuls test. **** *p* < 0.001; *** *p* < 0.001; ** *p* < 0.01; * *p* < 0.05. WT, wild-type; hMMP-2, human matrix metalloproteinase-2; SAL, saline; BLM, bleomycin; ns, not significant.

The total mRNA from the lungs of each mouse was extracted, and cDNA was synthesized by RT-PCT to evaluate the expression of anti-apoptotic and pro-apoptotic factors, mesenchymal markers, cytokines, and chemokines. The anti-apoptotic factors Bcl2, Bcl-XL, and BIRC4 were significantly decreased in the WT/BLM group compared to the WT/SAL group, but significantly increased in the hMMP-2 TG/BLM group compared to the WT/BLM group. In addition, the pro-apoptotic factor Bax was significantly increased in the WT/BLM group compared to the WT/SAL and hMMP-2 TG/BLM groups. No significant difference was observed in the mRNA expressions of BIRC1a, BIRC1b, BIRC2, BIRC3, BIRC5, BIRC6, and BIRC7 between WT/BLM and hMMP-2 TG/BLM groups (Figure 7). The protein levels of Bcl2 and Bax in plasma and BALF were also evaluated by enzyme immunoassays. The level of Bcl2 was significantly reduced, whereas the level of Bax was significantly increased in plasma and BALF from the WT/BLM group compared to the WT/SAL group (Supplementary Figure S7). The BALF level of Bcl2 was significantly increased and the plasma and BALF levels of Bax were significantly decreased in the hMMP-2/BLM group compared to the WT/BLM group. In addition, the Bcl2/Bax ratio in BALF was significantly increased in the hMMP-2/BLM group compared to the WT/BLM group (Supplementary Figure S7). Apoptosis plays an important role in the pathogenesis of lung fibrosis [56]. Therefore, the modulatory activity of pro-apoptotic and anti-apoptotic factors may be another explanation for the protective action of MMP-2 in pulmonary fibrosis.

**Figure 8:**
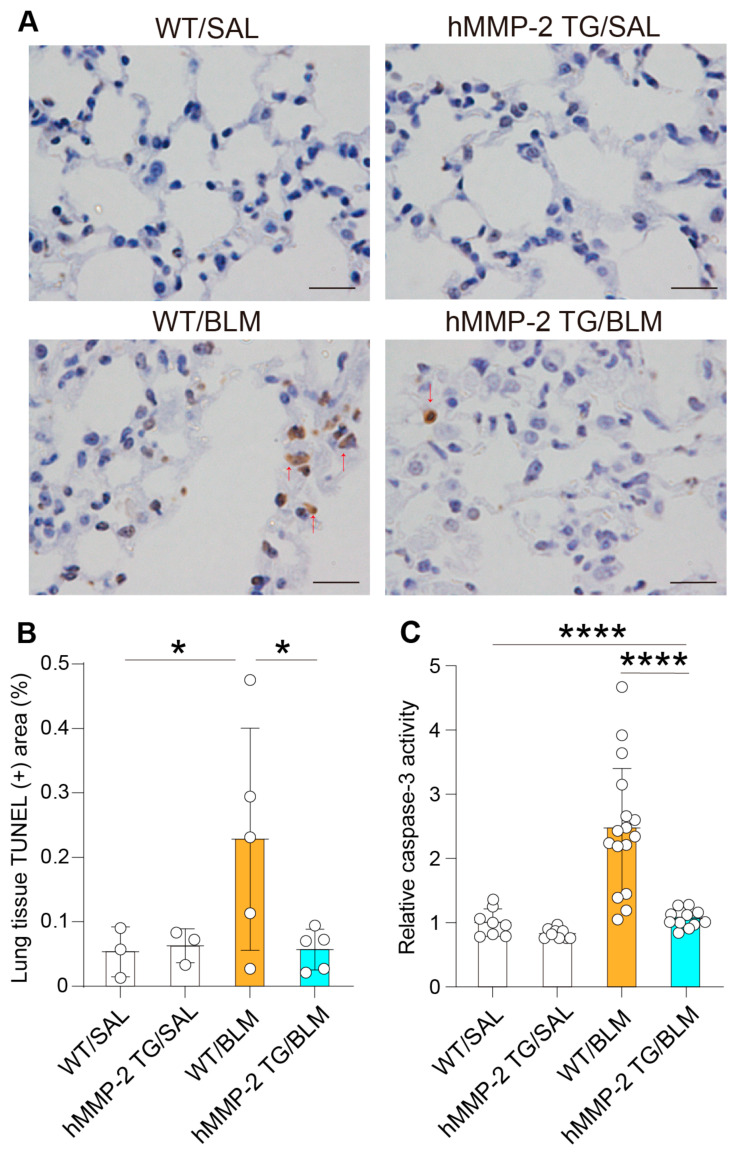
Reduced caspase-3 activity in mice with hMMP-2 overexpression. DNA fragmentation was evaluated by TUNEl assay, and the TUNEL-positive area was quantified using the WindRoof image processing software. Arrows indicate apoptotic cells (**A**,**B**). Caspase 3 activity was measured by a colorimetric assay (**C**). Data are the mean ± S.D. All mice were female. Statistical analysis by ANOVA with Newman-Keuls test. **** *p* < 0.0001; * *p* < 0.05; hM MP-2, human matrix metalloproteinase-2; SAL, saline; BLM, bleomycin.

Evaluation of DNA fragmentation showed a significantly enhanced TUNEL-positive area in the lung from the WT/BLM group compared to its hMMP-2/BLM counterpart (Figure 8A,B). In addition, a colorimetric assay using a substrate showed significantly increased caspase-3 activity in WT/BLM mice compared to WT/SAL and hMMP-2/BLM groups (Figure 8C). These observations further indicate the anti-apoptotic activity of hMMP-2 overexpression in BLM-induced lung fibrosis.

### 2.10. Active hMMP-2 Inhibits Apoptosis of Lung Epithelial Cells

**Figure 9:**
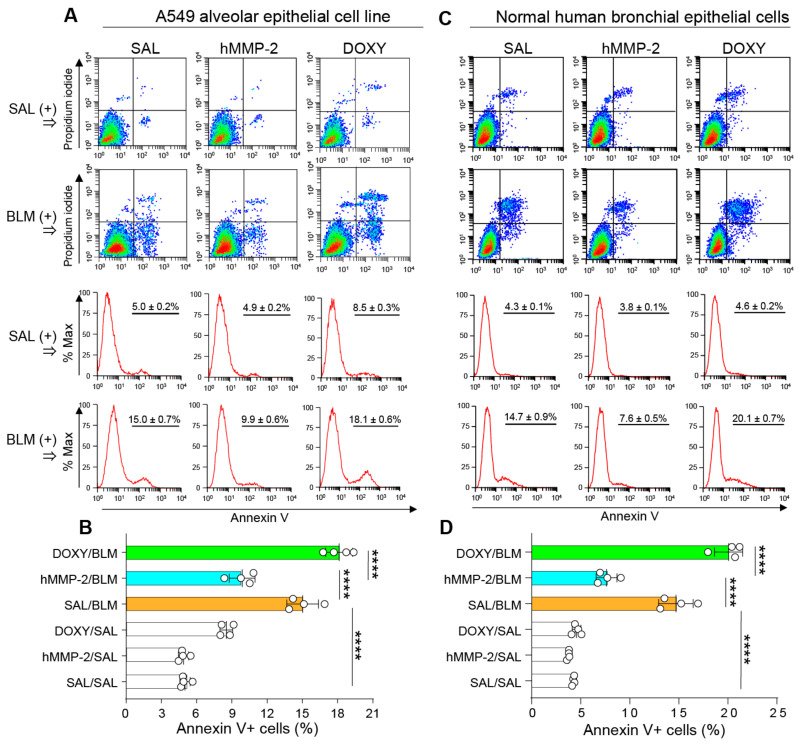
Active hMMP-2 inhibits apoptosis of lung epithelial cells. A549 cells (**A**,**B**) and normal human bronchial epithelial (NHBE) cells (**C**,**D**) were cultured and pretreated with active hMMP-2 (0.5 µg/mL) or doxycycline (25 µg/mL) before stimulating with BLM (50 mU/mL) to evaluate apoptosis by flow cytometry. n = 4 in each treatment group. Data are the mean ± S.D. Statistical analysis by ANOVA with Newman-Keuls test. **** *p* < 0.0001; hMMP-2, human matrix metalloproteinase-2; SAL, saline; BLM, bleomycin. DOXY, doxycycline.

To confirm the anti-apoptotic activity of MMP-2 on lung epithelial cells, A549 and NHBE cells were cultured and pretreated with active MMP-2 before stimulating with BLM to evaluate apoptosis by flow cytometry. Cells treated with active MMP-2 showed a significantly lower percentage of apoptotic cells than control cells (Figure 9A–D). In addition, doxycycline, a matrix metalloproteinase inhibitor, significantly blocked the suppression of BLM-induced apoptosis by active hMMP-2 (Figure 9A–D). The apoptotic effect of BLM on lung fibroblasts was also evaluated. BLM showed significant pro-apoptotic activity on lung fibroblasts, although the BLM’s apoptotic activity was weaker than that observed on lung epithelial cells. In addition, active MMP-2 could not inhibit the pro-apoptotic activity of BLM on lung fibroblasts (Supplementary Figure S8A,B). These findings demonstrate the direct inhibitory activity of MMP-2 on the apoptosis of lung epithelial cells.

### 2.11. Integrin-β3 Mediates the Inhibitory Activity of MMP-2 on Apoptosis of Lung Epithelial Cells

**Figure 10:**
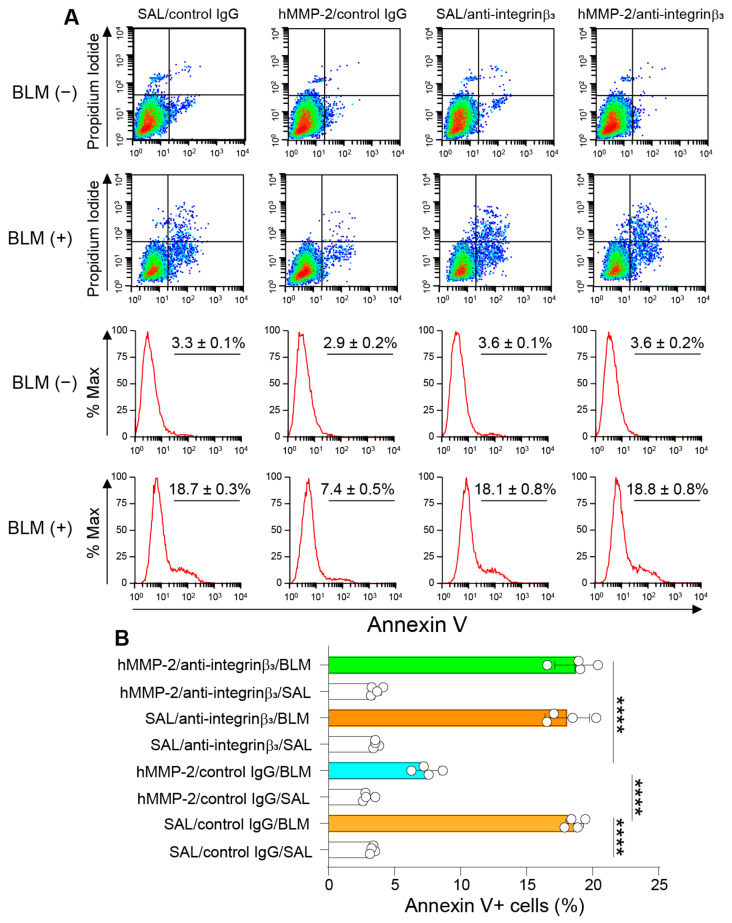
Anti-integrin-β3 antibody blocks the anti-apoptotic activity of hMMP-2 in lung epithelial cells. A549 cells were pretreated with 5 µg/mL of anti-integrin-β3 or control IgG for 30 min before adding 0.5 μg/mL hMMP-2. The cells were then treated with BLM (50 mU/mL) or saline and cultured for 24 h, and the number of apoptotic cells was evaluated by flow cytometry (**A**,**B**). Data are the mean ± S.D. Statistical analysis by ANOVA with Newman-Keuls test. **** *p* < 0.0001; hMMP-2, human matrix metalloproteinase-2; SAL, saline; BLM, bleomycin.

A previous study has shown that integrin-β3 mediates the protective activity of MMP-2 against apoptosis in pancreatic β-cells [57]. In agreement with this previous observation, we found that the inhibitory activity of active MMP-2 on apoptosis of A459 cells was significantly blocked in cells pretreated with the anti-integrin-β3 antibody, suggesting the involvement of integrin-β3 in MMP-2-mediated inhibition of lung epithelial apoptosis (Figure 10A,B).

## 3. Discussion

This study showed that hMMP-2 overexpression protects the lungs against BLM-induced inflammation and fibrosis.

Increased expression of hMMP-2 has been reported in pulmonary fibrosis, but whether it plays a pro-, antifibrotic, or no role remains unclear [58,59]. Bormann et al. found no difference in the severity of pulmonary fibrosis between WT and MMP-2 knockout mice that orotracheally received an adenoviral vector carrying the cDNA of active porcine TGFβ1 [42]. However, other studies have shown that MMP-2 may degrade epithelial cell basement membrane, leading to inflammatory exudation, angiogenesis, and fibro-proliferative responses, and that MMP-2 may promote epithelial–mesenchymal transition by inducing aberrant activation of the Wnt/β-catenin signaling pathway [60,61,62,63,64,65,66]. Therefore, we hypothesized that MMP-2 overexpression would accelerate lung fibrosis progression. To interrogate this hypothesis, we compared the grade of BLM-induced lung fibrosis between mice overexpressing hMMP-2 and WT counterparts. To our surprise, the MMP-2 TG mice showed significantly reduced lung inflammatory and fibrotic changes compared to WT mice, suggesting the protective function of MMP-2 in BLM-induced pulmonary fibrosis. Our research group previously reported these findings in an abstract form [67]. A previous study, showing that high lung tissue levels of MMP-2 associated with the administration of human umbilical cord endothelial cells reduce BLM-induced lung fibrosis, suggests the beneficial effects of MMP-2 in lung fibrosis [68]. Acceleration of experimental diabetic nephropathy in MMP-2 knockout mice and prevention of cardiac ventricular fibrosis after myocardial infarction by MMP-2 secretion from mesenchymal cells also indicate the protective activity of MMP-2 in organ fibrosis [69,70]. The different models of lung fibrosis used in the experiment may explain the discrepant results previously reported by Bormann et al. [42].

A question that needs to be addressed in the current study is whether the increased proteolysis of extracellular matrix (ECM) proteins can explain the protective activity of hMMP-2 overexpression against lung fibrosis. It was traditionally thought that the main function of MMPs is the proteolytic degradation of ECM and that aberrant organ fibrosis results from an imbalance between tissue deposition and the degradation of ECM proteins [63,71,72,73]. However, this theory had less support when several subsequent studies showed that the expression of the majority of MMPs is actually increased in pathological fibrotic tissues and that MMPs can directly affect cellular behavior and intracellular signaling pathways [21]. Indeed, in the present study, we found that hMMP-2 transgenic (TG) mice with lung fibrosis show significantly lower protein levels and mRNA expression of collagen and lower relative mRNA expression of fibronectin and α-SMC in the lungs than their wild-type (WT) counterparts, suggesting a possible direct effect of hMMP-2 on matrix protein production. Future in vitro studies using myofibroblasts may confirm this potential direct effect of hMMP-2 on collagen expression. However, under conditions with high concentrations and activity of MMP-2, the protective effect of high proteolytic activity against aberrant tissue fibrosis cannot be completely ruled out. The hMMP-2 TG mice expressed a high concentration of hMMP-2 and had high proteolytic activity in the lungs. Therefore, increased ECM degradation by hMMP-2 may also partly explain the reduction in lung fibrosis in our hMMP-2 TG mice.

Another alternative explanation for the beneficial effect of hMMP-2 in lung fibrosis is its anti-apoptotic activity on lung cells. Activation of programmed cell death signaling pathways has been reported in lung fibrosis [8]. Enhanced apoptosis of lung epithelial cells causes ineffective re-epithelization and ineffective macrophage-mediated removal of apoptotic cells, leading to sustained inflammation and tissue injury [74]. MMP-2 has been reported to inhibit the apoptosis of mesangial cells, endothelial cells, hepatocytes, and insulin-producing islet β-cells [57]. Furthermore, the protective activity of MMP-2 against β-cell death ameliorated hyperglycemia and enhanced insulin secretion and islet β-cell mass in an experimental animal model of diabetes mellitus. Previous studies have shown that MMP-2 interacts with integrin-β3 on the cell surface and activates the phosphatidylinositol-3′ kinase pathway, leading to suppression of cell apoptosis by decreasing the expression of Bax, a pro-apoptotic protein, and by increasing the expression of inhibitors of apoptosis proteins or BIRC proteins [57,75,76]. In agreement with these observations, in the present study, the lungs of MMP-2 TG mice treated with BLM showed significantly decreased expression of Bax, increased expression of the potent anti-apoptotic protein BIRC4, and reduced caspase-3 activity compared to their WT counterparts. In addition, an anti-integrin-β3 antibody suppressed the inhibitory activity of active hMMP-2 on BLM-induced apoptosis of A549 alveolar epithelial cells, suggesting a role for the integrin-β3/phosphatidylinositol-3′ kinase pathway in the inhibitory activity of MMP-2 in BLM-induced lung fibrosis.

Another possible mechanism for the protective activity of MMP-2 in lung fibrosis is the inhibition of the inflammatory response [77,78]. MMP-2 may dampen the recruitment of inflammatory cells by cleaving and inactivating the chemokines CCL7 (MCP-3) and CXCL12 (stromal cell-derived factor-1) [79,80,81]. The peptides released after CCL7 degradation by MMP-2 retain their binding capability to the chemokine receptor; therefore, they can act as chemokine receptor antagonists by inhibiting cell migration and inflammation [82]. MMP-2 also protects against allergic inflammation. MMP-2 promotes the trafficking and egression of parenchymal inflammatory cells into the airway lumen by establishing a chemotactic gradient in the lungs [83,84]. This MMP-2-mediated enhancement of allergic inflammatory cell clearance from the lungs prevents fatal asphyxiation [83,84]. MMP-2 may also stimulate the differentiation of macrophages to the M1 phenotype to inhibit allergic inflammation [85]. Consistent with the modulatory activity of MMP-2 on lung inflammation, the present study showed significantly fewer markers of inflammation (inflammatory cells, total protein) and reduced levels of inflammatory cytokines (IFNγ, TNFα, IL-6, osteopontin, IL-1β) and chemokines (MCP-1, MCP-3) in hMMP-2 TG mice treated with BLM compared to their WT counterparts. Inhibition of lung inflammation has been associated with the improvement of lung fibrosis [86]. In agreement, we found a significant correlation between inflammatory and fibrosis markers. Overall, these anti-inflammatory activities may explain the beneficial effects of MMP-2 in pulmonary fibrosis.

An abnormal accumulation of myofibroblasts in the lungs is a characteristic observation in lung fibrosis [1,2]. Chemokines stimulate the migration of fibroblasts/myofibroblasts to the lungs [1,2,9]. Myofibroblasts are the main secretors of extracellular matrix proteins, including collagen, vimentin, α-SMA, and fibronectin [1,2]. A mechanism contributing to an increased accumulation of myofibroblasts in the lungs is epithelial–mesenchymal transition (EMT) of alveolar epithelial cells [87]. Some MMPs (MMP-3 and MMP-7) have been reported to induce EMT in lung fibrosis [88]; however, the role of MMP-2 in EMT is unclear. Growth factors, including TGFβ1 and CTGF, are important promoters of EMT [89,90]. Aside from inducing EMT of alveolar epithelial cells, CTGF may also contribute to tissue fibrosis by increasing the production of matrix proteins, such as collagen, fibronectin, and α-SMA [91]. In the present study, mice of the hMMP-2 TG/BLM group showed significantly decreased lung levels of CTGF and reduced expression of collagen, fibronectin, and α-SMA compared to the WT/BLM group. Based on this observation, it is possible to speculate that, in our mouse model, MMP-2 indirectly reduced EMT and matrix protein production by inhibiting the expression of CTGF. Another explanation for myofibroblast accumulation in lung fibrosis is the enhanced expression of PDGF, a growth factor with potent mitogenic activity on fibroblasts and myofibroblasts [92]. The significantly decreased BALF level of PDGF in mice of the hMMP-2 TG/BLM group compared to the WT/BLM group, observed in the current study, suggests that MMP-2 may also ameliorate lung fibrosis by regulating the expression of PDGF. Overall, this suppressive activity on CTGF and PDGF expression may also explain, at least in part, the beneficial effects of MMP-2 in pulmonary fibrosis.

The present study has some limitations. The apoptosis-focused approach, the use of a TG mouse overexpressing hMMP-2 in several organs, and the lack of experiments to clarify the effect of hMMP-2 on matrix protein expression from myofibroblasts are limitations of this study. However, this is the first study to show that hMMP-2 ameliorates BLM-induced lung fibrosis by inhibiting apoptosis of lung epithelial cells. Further studies are warranted to confirm these findings, using mice with lung-specific overexpression of hMMP-2, and to clarify the effect of hMMP-2 on myofibroblasts’ profibrotic activity.

## 4. Materials and Methods

### 4.1. Animals

The characterization of human proMMP-2 transgenic (TG) mice in a C57BL/6 background has been previously reported [57,93]. Briefly, the full length of the human proMMP-2 cDNA was prepared and inserted into a vector under the CAG (cytomegalovirus enhancer, first exon and first intron of chicken β -actin gene, splice acceptor of rabbit β-globin gene) promoter, and then microinjected into fertilized eggs from C57BL/6J mice. The CAG promoter drives ubiquitous gene expression in various tissues [57]. The hMMP-2 TG mice express the transgene in all organs, including the lungs [57]. The hMMP-2 mice were born in the expected Mendelian ratio, indicating that hMMP-2 overexpression caused no embryonic lethality. The hMMP-2 mice were fertile with normal pregnancies and developed normally without structural or physiological lung abnormalities [57,93]. The reported blood concentration of hMMP-2 in the TG mice is 67 ng/mL [57].

Male mice develop far more severe bleomycin-induced lung fibrosis than their female counterparts [94]. However, regardless of sex, all mice develop lung fibrosis after systemic BLM infusion [94]. In the current study, we used female mice because they are less aggressive and easier to handle. Wild-type (WT) C57BL/6J mice were obtained from Nihon SLC (Hamamatsu, Japan). WT and hMMP-2 TG mice weighing 20~23 g and eight weeks of age were used in the experiments. The WT and hMMP-2 TG mice were housed in a specific pathogen-free condition at a relative humidity of about 60%, with a temperature of 21 °C, and under a constant 12 h light/dark cycle in the Mie University Experimental Animal Center. The mice were in a plastic cage supplied with wood-wool nesting material and free access to water and food (CLEA Japan Incorporation). Genotyping was performed by standard PCR analysis, using DNA was isolated from the tail of mice and specific primer pairs.

### 4.2. Experimental Design

The pulmonary fibrosis model was induced using bleomycin (BLM) (Nihon Kayaku, Tokyo, Japan). The mice were implanted in the back with subcutaneous osmotic minipumps (Alzet, model 2001; Durect Corporation, Cupertino, CA, USA) containing BLM (total dose of 100 mg/kg mouse weight dissolved in 200 µL saline). The osmotic minipump continuously delivers BLM at a 1 µL/hour rate for seven days. This experimental protocol was used because this dose of BLM (100 mg/kg) or even higher (150 mg/kg) was reported to induce progressive lung fibrosis with no mortality [95,96,97]. Control mice received saline through subcutaneous osmotic minipumps. The WT and hMMP-2 TG mice were allocated to the following treatment groups: wild-type (WT) mice treated with saline (SAL) (WT/SAL, n = 8) or BLM (WT/BLM, n = 16), and hMMP-2 TG mice that received saline (hMMP-2/SAL, n = 9) or BLM (hMMP-2 TG/BLM, n = 11). We followed the guidelines for animal investigations of ARRIVE (Animal Research: Reporting of In Vivo Experiments). Randomization of the mice was performed, and parameters were determined by researchers blinded to treatment groups.

### 4.3. Micro-Computed Tomography

A micro-computed tomography (CT) Latheta LCT-200 from Hitachi Aloka Medical (Tokyo, Japan) was used for a CT scan after anesthetizing the mice with 3% isoflurane inhalation and placing them in a prone position. In addition, the radiological findings of lung fibrosis were evaluated using an in-house CT fibrosis score previously described [98]. The lung volumes were calculated with CT values between −900 and 150 Hounsfield units within the thoracic cavity using the La Theta software version 3.30 (Hitachi-Aloka Medical Ltd., Tokyo, Japan).

### 4.4. Collection of Bronchoalveolar Lavage Fluid and Peripheral Blood

Bronchoalveolar lavage fluid (BALF) was collected under profound anesthesia with 3% isoflurane. The BALF was centrifuged (1000× *g*, 10 min, 4 °C) to separate the cells [99]. After centrifugation, the BALF supernatant was kept at −80 °C until use. First, the total number of cells was counted using a nucleocounter from ChemoMetec (Allerod, Denmark). BALF cells were then separated using a cytospin and stained with a May-Grünwald Giemsa stain (Merk, Darmstadt, Germany) for differential cell count.

### 4.5. Collection of Lung Samples

The lungs were resected after euthanasia by an overdose of 5% isoflurane for more than 60 s. Death was confirmed by cardiorespiratory arrest. One of the lungs was then fixed in formalin and immediately sent to Kinkiyoken Corporation (Otsu City, Shiga, Japan) for paraffin embedding and staining with hematoxylin-eosin and Masson trichrome. The Olympus BX50 microscope with a Plan objective and an Olympus DP70 digital camera (Tokyo, Japan) were used to evaluate histopathological findings. First, the grade of lung fibrosis was assessed using the Ashcroft fibrosis score [100]. For this purpose, several microphotographs (8 slides) covering almost all microscopic fields (5× magnification) of the lung of each mouse were taken, and then seven observers blinded for the treatment groups scored the fibrosis grade. The individual mean fibrosis score was then calculated. The degree of collagen deposition was evaluated in lung tissue stained with Masson trichrome. Several microphotographs covering all microscopic fields of the lung of each mouse were taken. The collagen volume fraction, the percentage of collagen-positive area per total tissue area, was calculated using the WinRoof image processing software (Mitani Corp., Fukui, Japan). These volume fractions were then used to calculate the total lung collagen volume fraction, using the mouse total lung volume measured with the microCT scan.

### 4.6. Cell Culture

The human lung adenocarcinoma A549 cell line was obtained from American Type Culture Collection (Manassas, VA, USA), and the primary (non-immortalized) normal human bronchial epithelial (NHBE) cells were from Clonetics (Walkersville, MD, USA). The cells were cultured in Dulbecco’s Modified Eagle Medium (DMEM) supplemented with 10% fetal calf serum, L-glutamine, and sodium pyruvate. Fibroblasts were prepared from the lungs of C57BL/6 WT mice by collagenase digestion. Fibroblasts were cultured in DMEM supplemented with 20% heat-inactivated fetal calf serum, 50 μg/mL penicillin, and 50 μg/mL streptomycin.

### 4.7. Evaluation of Apoptosis

Apoptosis in lung tissue was also evaluated by a TUNEL assay performed at MorphoTechnology Corporation (Sapporo, Hokkaido, Japan). To assess apoptosis in A549 and NHBE epithelial cells and mouse lung fibroblasts, subconfluent cells were serum-starved and then cultured for 48 h in the presence of BLM (50 mU/mL), active MMP-2 (0.5 µg/mL), or saline. A previous study has shown that 50 mU/mL of BLM significantly induces apoptosis of lung epithelial cells in vitro; thus, we used this concentration in our experiment [101]. A group of cells was pretreated with doxycycline (10 µg/mL), an inhibitor of matrix metalloproteinases, for 30 min before adding active hMMP-2 to the cells. Fluorescein-labeled annexin V and propidium iodide (FITC Annexin V Apoptosis Detection Kit with PI, Biolegend, San Diego, CA, USA) were used to evaluate apoptosis by flow cytometry (FACScan, BD Biosciences, Oxford, UK) [102]. A colorimetric assay based on the hydrolysis of the peptide substrate acetyl-Asp-Glu-Val-Asp p-nitroanilide (Ac-DEVD-pNA) was used to evaluate caspase-3 activity in lung samples (LKT Laboratories, Inc., St. Paul, MN, USA).

### 4.8. Biochemical Analysis

The total protein concentration in BALF was measured by a dye-binding assay using a commercial kit from ThermoFisher Scientific Incorporation (Waltham, MA, USA). The levels of TGFβ1, IL-1β, osteopontin, human MMP-2 (R&D Systems, Minneapolis, MN, USA), MCP-1, IL-13, interferon (IFN)-γ, IL-6 (BD Bioscience, BD opt-EIA kits, San Diego, CA, USA), MCP-3 (Preprotec, Rocky Hill, NJ, USA), connective tissue growth factor (CTGF) (Abgent, San Diego, CA, USA), Bax (LSBio, Seattle, WA, USA), and Bcl-2 (RayBiotech Life, inc, Peachtree Corners, GA, USA) were determined using commercial immunoassay kits according to the manufacturer’s instructions. Collagen type I was determined by enzyme immunoassay using an anti-collagen type I antibody and an anti-collagen type I biotin-conjugated antibody from Rockland Immunochemicals Incorporation (Limerick, PA, USA) following the manufacturer’s instructions. The level of total PDGF was measured by immunoassay as previously described [103]. The lung hydroxyproline content was determined by a colorimetric method using a commercial kit (Hydroxyproline colorimetric assay kit, BioVision, San Francisco, CA, USA) according to the manufacturer’s instructions.

### 4.9. Zymography and Western Blotting

The gelatinolytic activity of secreted hMMP-2 was analyzed by zymography on gelatin-containing polyacrylamide gels as described [95,104]. Western blotting was carried out following standard methods and using a commercially available antibody against TGFβ1 (R&D Systems, Minneapolis, MN, USA).

### 4.10. Analysis of Gene Expression

Analyses of gene expressions were performed by RT-PCR. Total RNA was extracted from the lung tissue by Trizol Reagent (Invitrogen, Carlsbad, CA, USA) and then subjected to reverse transcription (RT) using the Superscript Reverse Transcriptase (Invitrogen) and oligo-dT primers. Then, the cDNA was amplified using gene-specific primer pairs and the Quick Taq HS Dye Mix (TOYOBO, Osaka, Japan) [102]. Primer sequences are described in Supplementary Table S2. PCR reactions were optimized to 94 °C for 3 min, 30–35 amplification cycles at 94 °C for 10 s, 60 °C for 20 s, 72 °C for 40 s, and a final extension of 1 min at 72 °C. The products were run on a 2% agarose gel, and the bands were visualized by ultraviolet transillumination following ethidium bromide staining. All gene expressions were normalized by the glyceraldehyde 3-phosphate dehydrogenase (GAPDH) transcription level.

### 4.11. Statistical Analysis

Data were expressed as the mean ± standard deviation (S.D.). Statistical analyses were carried out by one-way analysis of variance (ANOVA) with the Newman-Keuls test or the Kruskal-Wallis ANOVA with Dunn’s test. GraphPad Prism version 5.0 was used for the statistical analysis (GraphPad Software, San Diego, CA, USA; http://www.graphpad.com/ (accessed on 3 February 2023). The strength of the relationship between parameters was evaluated by Spearman’s correlation. A *p* < 0.05 was considered statistically significant. We used the free downloadable software G Power to determine the sample size [105].

## 5. Conclusions

This study found that mice with lung overexpression of hMMP-2 had a reduction in the infiltration of inflammatory cells, expression of inflammatory and fibrotic cytokines, total lung collagen volume fraction, and apoptotic cells in the lungs compared to normal WT mice. These observations suggest a protective activity of MMP-2 and the potential therapeutic application of hMMP-2 in the clinic against pulmonary fibrosis. However, further studies should be conducted to clarify whether these mechanisms can conclusively explain the anti-fibrotic effect of hMMP-2.

## 6. Patents

E.C.G. and T.K. have a patent (WO2015087916A1) on the human MMP-2 TG mouse reported in this study.

## Data Availability

The data reported in the present study are available in the article. The corresponding author can provide the raw data upon request.

## Supplementary Materials

The following supporting information can be downloaded at: https://www.mdpi.com/article/10.3390/ijms24076695/s1.

## References

[1] Lederer D.J., Martinez F.J. (2018). Idiopathic Pulmonary Fibrosis. N. Engl. J. Med..

[2] Richeldi L., Collard H.R., Jones M.G. (2017). Idiopathic pulmonary fibrosis. Lancet.

[3] Raghu G., Chen S.Y., Yeh W.S., Maroni B., Li Q., Lee Y.C., Collard H.R. (2014). Idiopathic pulmonary fibrosis in US Medicare beneficiaries aged 65 years and older: Incidence, prevalence, and survival, 2001–2011. Lancet Respir. Med..

[4] Raghu G., Weycker D., Edelsberg J., Bradford W.Z., Oster G. (2006). Incidence and prevalence of idiopathic pulmonary fibrosis. Am. J. Respir. Crit. Care Med..

[5] Hutchinson J., Fogarty A., Hubbard R., McKeever T. (2015). Global incidence and mortality of idiopathic pulmonary fibrosis: A systematic review. Eur. Respir. J..

[6] Du Bois R.M. (2012). An earlier and more confident diagnosis of idiopathic pulmonary fibrosis. Eur. Respir. Rev..

[7] Baratella E., Ruaro B., Giudici F., Wade B., Santagiuliana M., Salton F., Confalonieri P., Simbolo M., Scarpa A., Tollot S. (2021). Evaluation of correlations between genetic variants and high-resolution computed tomography patterns in idiopathic pulmonary fibrosis. Diagnostics.

[8] Calabrese F., Lunardi F., Tauro V., Pezzuto F., Fortarezza F., Vedovelli L., Faccioli E., Balestro E., Schiavon M., Esposito G. (2022). RNA sequencing of epithelial cell/fibroblastic foci sandwich in idiopathic pulmonary fibrosis: New insights on the signaling pathway. Int. J. Mol. Sci..

[9] Veres-Székely A., Pap D., Szebeni B., Őrfi L., Szász C., Pajtók C., Lévai E., Szabó A.J., Vannay Á. (2022). Transient agarose spot (TAS) assay: A new method to investigate cell migration. Int. J. Mol. Sci..

[10] Shimizu S., Gabazza E.C., Ogawa T., Tojima I., Hoshi E., Kouzaki H., Shimizu T. (2011). Role of thrombin in chronic rhinosinusitis-associated tissue remodeling. Am. J. Rhinol. Allergy.

[11] Liu G., Philp A.M., Corte T., Travis M.A., Schilter H., Hansbro N.G., Burns C.J., Eapen M.S., Sohal S.S., Burgess J.K. (2021). Therapeutic targets in lung tissue remodelling and fibrosis. Pharmacol. Ther..

[12] Gabazza E.C., Taguchi O., Kamada H., Hayashi T., Adachi Y., Suzuki K. (2004). Progress in the understanding of protease-activated receptors. Int. J. Hematol..

[13] Fukuda Y., Mochimaru H., Terasaki Y., Kawamoto M., Kudoh S. (2001). Mechanism of structural remodeling in pulmonary fibrosis. Chest.

[14] Hayashi T., Stetler-Stevenson W.G., Fleming M.V., Fishback N., Koss M.N., Liotta L.A., Ferrans V.J., Travis W.D. (1996). Immunohistochemical study of metalloproteinases and their tissue inhibitors in the lungs of patients with diffuse alveolar damage and idiopathic pulmonary fibrosis. Am. J. Pathol..

[15] Maatta M., Laurila H.P., Holopainen S., Aaltonen K., Lilja-Maula L., Viitanen S., Rajamaki M.M. (2021). Matrix metalloproteinase-2, -7, and -9 activities in dogs with idiopathic pulmonary fibrosis compared to healthy dogs and dogs with other respiratory diseases. J. Vet. Intern. Med..

[16] Suga M., Iyonaga K., Okamoto T., Gushima Y., Miyakawa H., Akaike T., Ando M. (2000). Characteristic elevation of matrix metalloproteinase activity in idiopathic interstitial pneumonias. Am. J. Respir. Crit. Care Med..

[17] Summer R., Krishna R., Schriner D., Cuevas-Mora K., Sales D., Para R., Roman J., Nieweld C., Gochuico B.R., Romero F. (2019). Matrix metalloproteinase activity in the lung is increased in Hermansky-Pudlak syndrome. Orphanet J. Rare Dis..

[18] Todd J.L., Vinisko R., Liu Y., Neely M.L., Overton R., Flaherty K.R., Noth I., Newby L.K., Lasky J.A., Olman M.A. (2020). Circulating matrix metalloproteinases and tissue metalloproteinase inhibitors in patients with idiopathic pulmonary fibrosis in the multicenter IPF-PRO Registry cohort. BMC Pulm. Med..

[19] Chulia-Peris L., Carreres-Rey C., Gabasa M., Alcaraz J., Carretero J., Pereda J. (2022). Matrix Metalloproteinases and Their Inhibitors in Pulmonary Fibrosis: EMMPRIN/CD147 Comes into Play. Int. J. Mol. Sci..

[20] Mahalanobish S., Saha S., Dutta S., Sil P.C. (2020). Matrix metalloproteinase: An upcoming therapeutic approach for idiopathic pulmonary fibrosis. Pharmacol. Res..

[21] Pardo A., Cabrera S., Maldonado M., Selman M. (2016). Role of matrix metalloproteinases in the pathogenesis of idiopathic pulmonary fibrosis. Respir. Res..

[22] Aoyagi-Ikeda K., Maeno T., Matsui H., Ueno M., Hara K., Aoki Y., Aoki F., Shimizu T., Doi H., Kawai-Kowase K. (2011). Notch induces myofibroblast differentiation of alveolar epithelial cells via transforming growth factor-beta-Smad3 pathway. Am. J. Respir. Cell Mol. Biol..

[23] Craig V.J., Quintero P.A., Fyfe S.E., Patel A.S., Knolle M.D., Kobzik L., Owen C.A. (2013). Profibrotic activities for matrix metalloproteinase-8 during bleomycin-mediated lung injury. J. Immunol..

[24] Garcia-Prieto E., Gonzalez-Lopez A., Cabrera S., Astudillo A., Gutierrez-Fernandez A., Fanjul-Fernandez M., Batalla-Solis E., Puente X.S., Fueyo A., Lopez-Otin C. (2010). Resistance to bleomycin-induced lung fibrosis in MMP-8 deficient mice is mediated by interleukin-10. PLoS ONE.

[25] Gharib S.A., Johnston L.K., Huizar I., Birkland T.P., Hanson J., Wang Y., Parks W.C., Manicone A.M. (2014). MMP28 promotes macrophage polarization toward M2 cells and augments pulmonary fibrosis. J. Leukoc. Biol..

[26] Jara P., Calyeca J., Romero Y., Placido L., Yu G., Kaminski N., Maldonado V., Cisneros J., Selman M., Pardo A. (2015). Matrix metalloproteinase (MMP)-19-deficient fibroblasts display a profibrotic phenotype. Am. J. Physiol. Lung Cell. Mol. Physiol..

[27] Rohani M.G., McMahan R.S., Razumova M.V., Hertz A.L., Cieslewicz M., Pun S.H., Regnier M., Wang Y., Birkland T.P., Parks W.C. (2015). MMP-10 Regulates Collagenolytic Activity of Alternatively Activated Resident Macrophages. J. Invest. Dermatol..

[28] Yamashita C.M., Dolgonos L., Zemans R.L., Young S.K., Robertson J., Briones N., Suzuki T., Campbell M.N., Gauldie J., Radisky D.C. (2011). Matrix metalloproteinase 3 is a mediator of pulmonary fibrosis. Am. J. Pathol..

[29] Yu G., Kovkarova-Naumovski E., Jara P., Parwani A., Kass D., Ruiz V., Lopez-Otin C., Rosas I.O., Gibson K.F., Cabrera S. (2012). Matrix metalloproteinase-19 is a key regulator of lung fibrosis in mice and humans. Am. J. Respir. Crit. Care Med..

[30] Cabrera S., Maciel M., Hernandez-Barrientos D., Barrientos D., Calyeca J., Gaxiola M., Selman M., Pardo A. (2019). Delayed resolution of bleomycin-induced pulmonary fibrosis in absence of MMP13 (collagenase 3). Am. J. Physiol. Lung Cell. Mol. Physiol..

[31] Checa M., Ruiz V., Montano M., Velazquez-Cruz R., Selman M., Pardo A. (2008). MMP-1 polymorphisms and the risk of idiopathic pulmonary fibrosis. Hum. Genet..

[32] Flechsig P., Hartenstein B., Teurich S., Dadrich M., Hauser K., Abdollahi A., Grone H.J., Angel P., Huber P.E. (2010). Loss of matrix metalloproteinase-13 attenuates murine radiation-induced pulmonary fibrosis. Int. J. Radiat. Oncol. Biol. Phys..

[33] Gabasa M., Arshakyan M., Llorente A., Chulia-Peris L., Pavelescu I., Xaubet A., Pereda J., Alcaraz J. (2020). Interleukin-1beta modulation of the mechanobiology of primary human pulmonary fibroblasts: Potential implications in lung repair. Int. J. Mol. Sci..

[34] Herrera I., Cisneros J., Maldonado M., Ramirez R., Ortiz-Quintero B., Anso E., Chandel N.S., Selman M., Pardo A. (2013). Matrix metalloproteinase (MMP)-1 induces lung alveolar epithelial cell migration and proliferation, protects from apoptosis, and represses mitochondrial oxygen consumption. J. Biol. Chem..

[35] Manicone A.M., Huizar I., McGuire J.K. (2009). Matrilysin (Matrix Metalloproteinase-7) regulates anti-inflammatory and antifibrotic pulmonary dendritic cells that express CD103 (alpha(E)beta(7)-integrin). Am. J. Pathol..

[36] Nkyimbeng T., Ruppert C., Shiomi T., Dahal B., Lang G., Seeger W., Okada Y., D’Armiento J., Gunther A. (2013). Pivotal role of matrix metalloproteinase 13 in extracellular matrix turnover in idiopathic pulmonary fibrosis. PLoS ONE.

[37] Sen A.I., Shiomi T., Okada Y., D’Armiento J.M. (2010). Deficiency of matrix metalloproteinase-13 increases inflammation after acute lung injury. Exp. Lung Res..

[38] Xiong Y., Zhang J., Shi L., Ning Y., Zhu Y., Chen S., Yang M., Chen J., Zhou G.W., Li Q. (2017). NOGO-B promotes EMT in lung fibrosis via MMP14 mediates free TGF-β1 formation. Oncotarget.

[39] Zigrino P., Brinckmann J., Niehoff A., Lu Y., Giebeler N., Eckes B., Kadler K.E., Mauch C. (2016). Fibroblast-derived MMP-14 regulates collagen homeostasis in adult skin. J. Investig. Dermatol..

[40] Zuo F., Kaminski N., Eugui E., Allard J., Yakhini Z., Ben-Dor A., Lollini L., Morris D., Kim Y., DeLustro B. (2002). Gene expression analysis reveals matrilysin as a key regulator of pulmonary fibrosis in mice and humans. Proc. Natl. Acad. Sci. USA.

[41] Betsuyaku T., Fukuda Y., Parks W.C., Shipley J.M., Senior R.M. (2000). Gelatinase B is required for alveolar bronchiolization after intratracheal bleomycin. Am. J. Pathol..

[42] Bormann T., Maus R., Stolper J., Tort Tarres M., Brandenberger C., Wedekind D., Jonigk D., Welte T., Gauldie J., Kolb M. (2022). Role of matrix metalloprotease-2 and MMP-9 in experimental lung fibrosis in mice. Respir. Res..

[43] Cabrera S., Gaxiola M., Arreola J.L., Ramirez R., Jara P., D’Armiento J., Richards T., Selman M., Pardo A. (2007). Overexpression of MMP9 in macrophages attenuates pulmonary fibrosis induced by bleomycin. Int. J. Biochem. Cell Biol..

[44] Espindola M.S., Habiel D.M., Coelho A.L., Stripp B., Parks W.C., Oldham J., Martinez F.J., Noth I., Lopez D., Mikels-Vigdal A. (2021). Differential Responses to Targeting Matrix Metalloproteinase 9 in Idiopathic Pulmonary Fibrosis. Am. J. Respir. Crit. Care Med..

[45] Hu B., Wu Z., Bai D., Tang R., Phan S. (2012). Matrix metalloproteinase-12 (MM12) inhibits myofibroblasts differentiation and lung fibrosis. FASEB J..

[46] Kang H.R., Cho S.J., Lee C.G., Homer R.J., Elias J.A. (2007). Transforming growth factor (TGF)-beta1 stimulates pulmonary fibrosis and inflammation via a Bax-dependent, bid-activated pathway that involves matrix metalloproteinase-12. J. Biol. Chem..

[47] Kim T.H., Kim S.H., Seo J.Y., Chung H., Kwak H.J., Lee S.K., Yoon H.J., Shin D.H., Park S.S., Sohn J.W. (2011). Blockade of the Wnt/beta-catenin pathway attenuates bleomycin-induced pulmonary fibrosis. Tohoku J. Exp. Med..

[48] Manoury B., Nenan S., Guenon I., Boichot E., Planquois J.M., Bertrand C.P., Lagente V. (2006). Macrophage metalloelastase (MMP-12) deficiency does not alter bleomycin-induced pulmonary fibrosis in mice. J. Inflamm..

[49] Ramirez G., Hagood J.S., Sanders Y., Ramirez R., Becerril C., Segura L., Barrera L., Selman M., Pardo A. (2011). Absence of Thy-1 results in TGF-beta induced MMP-9 expression and confers a profibrotic phenotype to human lung fibroblasts. Lab. Investig..

[50] Xu L., Bian W., Gu X.H., Shen C. (2017). Genetic polymorphism in matrix metalloproteinase-9 and transforming growth factor-beta1 and susceptibility to combined pulmonary fibrosis and emphysema in a Chinese population. Kaohsiung J. Med. Sci..

[51] Dean R.A., Overall C.M. (2007). Proteomics discovery of metalloproteinase substrates in the cellular context by iTRAQ labeling reveals a diverse MMP-2 substrate degradome. Mol. Cell. Proteomics.

[52] Ito A., Mukaiyama A., Itoh Y., Nagase H., Thogersen I.B., Enghild J.J., Sasaguri Y., Mori Y. (1996). Degradation of interleukin 1beta by matrix metalloproteinases. J. Biol. Chem..

[53] Overall C.M., Dean R.A. (2006). Degradomics: Systems biology of the protease web. Pleiotropic roles of MMPs in cancer. Cancer Metastasis Rev..

[54] Dean R.A., Butler G.S., Hamma-Kourbali Y., Delbe J., Brigstock D.R., Courty J., Overall C.M. (2007). Identification of candidate angiogenic inhibitors processed by matrix metalloproteinase 2 (MMP-2) in cell-based proteomic screens: Disruption of vascular endothelial growth factor (VEGF)/heparin affin regulatory peptide (pleiotrophin) and VEGF/Connective tissue growth factor angiogenic inhibitory complexes by MMP-2 proteolysis. Mol. Cell. Biol..

[55] Madtes D.K., Elston A.L., Hackman R.C., Dunn A.R., Clark J.G. (1999). Transforming growth factor-alpha deficiency reduces pulmonary fibrosis in transgenic mice. Am. J. Respir. Cell Mol. Biol..

[56] Kim S.J., Cheresh P., Jablonski R.P., Williams D.B., Kamp D.W. (2015). The Role of Mitochondrial DNA in Mediating Alveolar Epithelial Cell Apoptosis and Pulmonary Fibrosis. Int. J. Mol. Sci..

[57] Nishihama K., Yasuma T., Yano Y., D’ Alessandro-Gabazza C.N., Toda M., Hinneh J.A., Baffour Tonto P., Takeshita A., Totoki T., Mifuji-Moroka R. (2018). Anti-apoptotic activity of human matrix metalloproteinase-2 attenuates diabetes mellitus. Metabolism.

[58] Fukuda Y., Ishizaki M., Kudoh S., Kitaichi M., Yamanaka N. (1998). Localization of matrix metalloproteinases-1, -2, and -9 and tissue inhibitor of metalloproteinase-2 in interstitial lung diseases. Lab. Investig..

[59] McKeown S., Richter A.G., O’Kane C., McAuley D.F., Thickett D.R. (2009). MMP expression and abnormal lung permeability are important determinants of outcome in IPF. Eur. Respir. J..

[60] Cheng S., Pollock A.S., Mahimkar R., Olson J.L., Lovett D.H. (2006). Matrix metalloproteinase 2 and basement membrane integrity: A unifying mechanism for progressive renal injury. FASEB J..

[61] Keane M.P., Arenberg D.A., Lynch J.P., Whyte R.I., Iannettoni M.D., Burdick M.D., Wilke C.A., Morris S.B., Glass M.C., DiGiovine B. (1997). The CXC chemokines, IL-8 and IP-10, regulate angiogenic activity in idiopathic pulmonary fibrosis. J. Immunol..

[62] Nguyen M., Arkell J., Jackson C.J. (2001). Human endothelial gelatinases and angiogenesis. Int. J. Biochem. Cell Biol..

[63] Ruiz V., Ordonez R.M., Berumen J., Ramirez R., Uhal B., Becerril C., Pardo A., Selman M. (2003). Unbalanced collagenases/TIMP-1 expression and epithelial apoptosis in experimental lung fibrosis. Am. J. Physiol. Lung Cell Mol. Physiol..

[64] Seomun Y., Kim J., Lee E.H., Joo C.K. (2001). Overexpression of matrix metalloproteinase-2 mediates phenotypic transformation of lens epithelial cells. Biochem. J..

[65] Song W., Jackson K., McGuire P.G. (2000). Degradation of type IV collagen by matrix metalloproteinases is an important step in the epithelial-mesenchymal transformation of the endocardial cushions. Dev. Biol..

[66] Van der Velden J.L., Guala A.S., Leggett S.E., Sluimer J., Badura E.C., Janssen-Heininger Y.M. (2012). Induction of a mesenchymal expression program in lung epithelial cells by wingless protein (Wnt)/beta-catenin requires the presence of c-Jun N-terminal kinase-1 (JNK1). Am. J. Respir. Cell Mol. Biol..

[67] Tomaru A., Gabazza E., Kobayashi T., Kobayashi H., Taguchi O., Takagi T., Oonishi M., Fujiwara K., D’Alessandro Gabazza C., Takahashi Y. (2015). Matrix metalloproteinase-2 is protective in bleomycin-induced pulmonary fibrosis. Eur. Respir. J..

[68] Moodley Y., Atienza D., Manuelpillai U., Samuel C.S., Tchongue J., Ilancheran S., Boyd R., Trounson A. (2009). Human umbilical cord mesenchymal stem cells reduce fibrosis of bleomycin-induced lung injury. Am. J. Pathol..

[69] Mias C., Lairez O., Trouche E., Roncalli J., Calise D., Seguelas M.H., Ordener C., Piercecchi-Marti M.D., Auge N., Salvayre A.N. (2009). Mesenchymal stem cells promote matrix metalloproteinase secretion by cardiac fibroblasts and reduce cardiac ventricular fibrosis after myocardial infarction. Stem Cells.

[70] Takamiya Y., Fukami K., Yamagishi S., Kaida Y., Nakayama Y., Obara N., Iwatani R., Ando R., Koike K., Matsui T. (2013). Experimental diabetic nephropathy is accelerated in matrix metalloproteinase-2 knockout mice. Nephrol. Dial. Transplant..

[71] Montano M., Ramos C., Gonzalez G., Vadillo F., Pardo A., Selman M. (1989). Lung collagenase inhibitors and spontaneous and latent collagenase activity in idiopathic pulmonary fibrosis and hypersensitivity pneumonitis. Chest.

[72] Montfort I., Perez-Tamayo R. (1978). Collagenase in experimental carbon tetrachloride cirrhosis of the liver. Am. J. Pathol..

[73] Selman M., Ruiz V., Cabrera S., Segura L., Ramirez R., Barrios R., Pardo A. (2000). TIMP-1, -2, -3, and -4 in idiopathic pulmonary fibrosis. A prevailing nondegradative lung microenvironment?. Am. J. Physiol. Lung Cell Mol. Physiol..

[74] Drakopanagiotakis F., Xifteri A., Polychronopoulos V., Bouros D. (2008). Apoptosis in lung injury and fibrosis. Eur. Respir. J..

[75] Chetty C., Bhoopathi P., Lakka S.S., Rao J.S. (2007). MMP-2 siRNA induced Fas/CD95-mediated extrinsic II apoptotic pathway in the A549 lung adenocarcinoma cell line. Oncogene.

[76] Silletti S., Kessler T., Goldberg J., Boger D.L., Cheresh D.A. (2001). Disruption of matrix metalloproteinase 2 binding to integrin alpha vbeta 3 by an organic molecule inhibits angiogenesis and tumor growth in vivo. Proc. Natl. Acad. Sci. USA.

[77] Manicone A.M., McGuire J.K. (2008). Matrix metalloproteinases as modulators of inflammation. Semin. Cell. Dev. Biol..

[78] Parks W.C., Wilson C.L., Lopez-Boado Y.S. (2004). Matrix metalloproteinases as modulators of inflammation and innate immunity. Nat. Rev. Immunol..

[79] McQuibban G.A., Butler G.S., Gong J.H., Bendall L., Power C., Clark-Lewis I., Overall C.M. (2001). Matrix metalloproteinase activity inactivates the CXC chemokine stromal cell-derived factor-1. J. Biol. Chem..

[80] McQuibban G.A., Gong J.H., Tam E.M., McCulloch C.A., Clark-Lewis I., Overall C.M. (2000). Inflammation dampened by gelatinase A cleavage of monocyte chemoattractant protein-3. Science.

[81] Zhang K., McQuibban G.A., Silva C., Butler G.S., Johnston J.B., Holden J., Clark-Lewis I., Overall C.M., Power C. (2003). HIV-induced metalloproteinase processing of the chemokine stromal cell derived factor-1 causes neurodegeneration. Nat. Neurosci..

[82] McQuibban G.A., Gong J.H., Wong J.P., Wallace J.L., Clark-Lewis I., Overall C.M. (2002). Matrix metalloproteinase processing of monocyte chemoattractant proteins generates CC chemokine receptor antagonists with anti-inflammatory properties in vivo. Blood.

[83] Corry D.B., Rishi K., Kanellis J., Kiss A., Song Lz L.Z., Xu J., Feng L., Werb Z., Kheradmand F. (2002). Decreased allergic lung inflammatory cell egression and increased susceptibility to asphyxiation in MMP2-deficiency. Nat. Immunol..

[84] Greenlee K.J., Corry D.B., Engler D.A., Matsunami R.K., Tessier P., Cook R.G., Werb Z., Kheradmand F. (2006). Proteomic identification of in vivo substrates for matrix metalloproteinases 2 and 9 reveals a mechanism for resolution of inflammation. J. Immunol..

[85] Takahashi Y., Kobayashi T., D’Alessandro-Gabazza C.N., Gabazza C.N., Toda M., Fujiwara K., Okano T., Fujimoto H., Asayama K., Takeshita A. (2019). Protective Role of Matrix Metalloproteinase-2 in Allergic Bronchial Asthma. Front. Immunol..

[86] Tanner L., Single A.B., Bhongir R.K.V., Heusel M., Mohanty T., Karlsson C.A.Q., Pan L., Clausson C.M., Bergwik J., Wang K. (2023). Small-molecule-mediated OGG1 inhibition attenuates pulmonary inflammation and lung fibrosis in a murine lung fibrosis model. Nat. Commun..

[87] Jolly M.K., Ward C., Eapen M.S., Myers S., Hallgren O., Levine H., Sohal S.S. (2018). Epithelial-mesenchymal transition, a spectrum of states: Role in lung development, homeostasis, and disease. Dev. Dyn..

[88] Craig V.J., Zhang L., Hagood J.S., Owen C.A. (2015). Matrix metalloproteinases as therapeutic targets for idiopathic pulmonary fibrosis. Am. J. Respir. Cell Mol. Biol..

[89] Shafieian M., Chen S., Wu S. (2015). Integrin-linked kinase mediates CTGF-induced epithelial to mesenchymal transition in alveolar type II epithelial cells. Pediatr. Res..

[90] Sonnylal S., Xu S., Jones H., Tam A., Sreeram V.R., Ponticos M., Norman J., Agrawal P., Abraham D., de Crombrugghe B. (2013). Connective tissue growth factor causes EMT-like cell fate changes in vivo and in vitro. J. Cell Sci..

[91] Lipson K.E., Wong C., Teng Y., Spong S. (2012). CTGF is a central mediator of tissue remodeling and fibrosis and its inhibition can reverse the process of fibrosis. Fibrogenesis Tissue Repair..

[92] Noskovičová N., Petřek M., Eickelberg O., Heinzelmann K. (2015). Platelet-derived growth factor signaling in the lung. From lung development and disease to clinical studies. Am. J. Respir. Cell Mol. Biol..

[93] Onishi M., Kobayashi T., D’Alessandro-Gabazza C.N., Fujimoto H., Chelakkot-Govindalayathil A.L., Takahashi Y., Yasuma T., Nishihama K., Toda M., Takei Y. (2018). Mice overexpressing latent matrix metalloproteinase-2 develop lung emphysema after short-term exposure to cigarette smoke extract. Biochem. Biophys. Res. Commun..

[94] Redente E.F., Jacobsen K.M., Solomon J.J., Lara A.R., Faubel S., Keith R.C., Henson P.M., Downey G.P., Riches D.W. (2011). Age and sex dimorphisms contribute to the severity of bleomycin-induced lung injury and fibrosis. Am. J. Physiol. Lung Cell. Mol. Physiol..

[95] Boveda-Ruiz D., D’Alessandro-Gabazza C.N., Toda M., Toda M., Takagi T., Naito M., Matsushima Y., Matsumoto T., Kobayashi T., Gil-Bernabe P. (2013). Differential role of regulatory T cells in early and late stages of pulmonary fibrosis. Immunobiology.

[96] Lemaire R., Burwell T., Sun H., Delaney T., Bakken J., Cheng L., Rebelatto M.C., Czapiga M., de-Mendez I., Coyle A.J. (2016). Resolution of Skin Fibrosis by Neutralization of the Antifibrinolytic Function of Plasminogen Activator Inhibitor 1. Arthritis Rheumatol..

[97] Watanabe T., Nishimoto T., Mlakar L., Heywood J., Malaab M., Hoffman S., Feghali-Bostwick C. (2017). Optimization of a murine and human tissue model to recapitulate dermal and pulmonary features of systemic sclerosis. PLoS ONE.

[98] D’Alessandro-Gabazza C.N., Kobayashi T., Yasuma T., Toda M., Kim H., Fujimoto H., Hataji O., Takeshita A., Nishihama K., Okano T. (2020). A Staphylococcus pro-apoptotic peptide induces acute exacerbation of pulmonary fibrosis. Nat. Commun..

[99] D’Alessandro-Gabazza C.N., Yasuma T., Kobayashi T., Toda M., Abdel-Hamid A.M., Fujimoto H., Hataji O., Nakahara H., Takeshita A., Nishihama K. (2022). Inhibition of lung microbiota-derived proapoptotic peptides ameliorates acute exacerbation of pulmonary fibrosis. Nat. Commun..

[100] Ashcroft T., Simpson J.M., Timbrell V. (1988). Simple method of estimating severity of pulmonary fibrosis on a numerical scale. J. Clin. Pathol..

[101] Wallach-Dayan S.B., Izbicki G., Cohen P.Y., Gerstl-Golan R., Fine A., Breuer R. (2006). Bleomycin initiates apoptosis of lung epithelial cells by ROS but not by Fas/FasL pathway. Am. J. Physiol. Lung Cell. Mol. Physiol..

[102] Deguchi H., Takeya H., Wada H., Gabazza E.C., Hayashi N., Urano H., Suzuki K. (1997). Dilazep, an antiplatelet agent, inhibits tissue factor expression in endothelial cells and monocytes. Blood.

[103] Shimizu S., Gabazza E.C., Hayashi T., Ido M., Adachi Y., Suzuki K. (2000). Thrombin stimulates the expression of PDGF in lung epithelial cells. Am. J. Physiol. Lung Cell. Mol. Physiol..

[104] Birkedal-Hansen H., Taylor R.E. (1982). Detergent-activation of latent collagenase and resolution of its component molecules. Biochem. Biophys. Res. Commun..

[105] Faul F., Erdfelder E., Lang A.G., Buchner A. (2007). G*Power 3, a flexible statistical power analysis program for the social, behavioral, and biomedical sciences. Behav. Res. Methods.

