# Supplementary material for: Amelioration of Pulmonary Fibrosis by Matrix Metalloproteinase-2 Overexpression
Source: Int J Mol Sci. 2023 Apr 3;24(7):6695. doi: 10.3390/ijms24076695 (PMC10095307; doi:10.3390/ijms24076695)
Supplement: Supplementary file 1 [file ijms-24-06695-s001.zip › ijms-2268307-supplementary.pdf]

Supplemental Information

Amelioration of Pulmonary Fibrosis by Matrix Metalloproteinase-2 Overexpression

Ryo Inoue, Taro Yasuma, Valeria Fridman D'Alessandro, Masaaki Toda, Toshiyuki Ito, Atsushi Tomaru, Corina N. D' Alessandro-Gabazza, Tatsuki Tsuruga, Tomohito Okano, Atsuro Takeshita, Kota Nishihama, Hajime Fujimoto, Tetsu Kobayashi, Esteban C. Gabazza.

Supplementary Table S1. Matrix metalloproteinases in pulmonary fibrosis

MMPs	Reference number
MMP-1	
Profibrotic	31
Antifibrotic	34
MMP-2	
Profibrotic	50
Antifibrotic	current study
No effect on fibrosis	42
MMP-3	
Profibrotic	28
MMP-7	
Profibrotic	40
Antifibrotic	35
MMP-8	
Profibrotic	23, 24
MMP-9	
Profibrotic	44, 48, 49
Antifibrotic	21, 43
No effect on collagen deposition	41, 42
MMP-10	
Antifibrotic	27
MMP-11	
Profibrotic	22
MMP-12	
Profibrotic	46
Antifibrotic	45
No effect on fibrosis	47
MMP-13	
Profibrotic	32
Antifibrotic	30, 36, 37
MMP-14	
Profibrotic	38
Antifibrotic	39
MMP-15, -16, -17	
Unknown	
MMP-19	
Antifibrotic	26, 29
MMP-20, -21, -22, -23, -24, -25, -26, -27	
Unknown	
MMP-28	
Profibrotic	25

MMP, matrix metalloproteinase

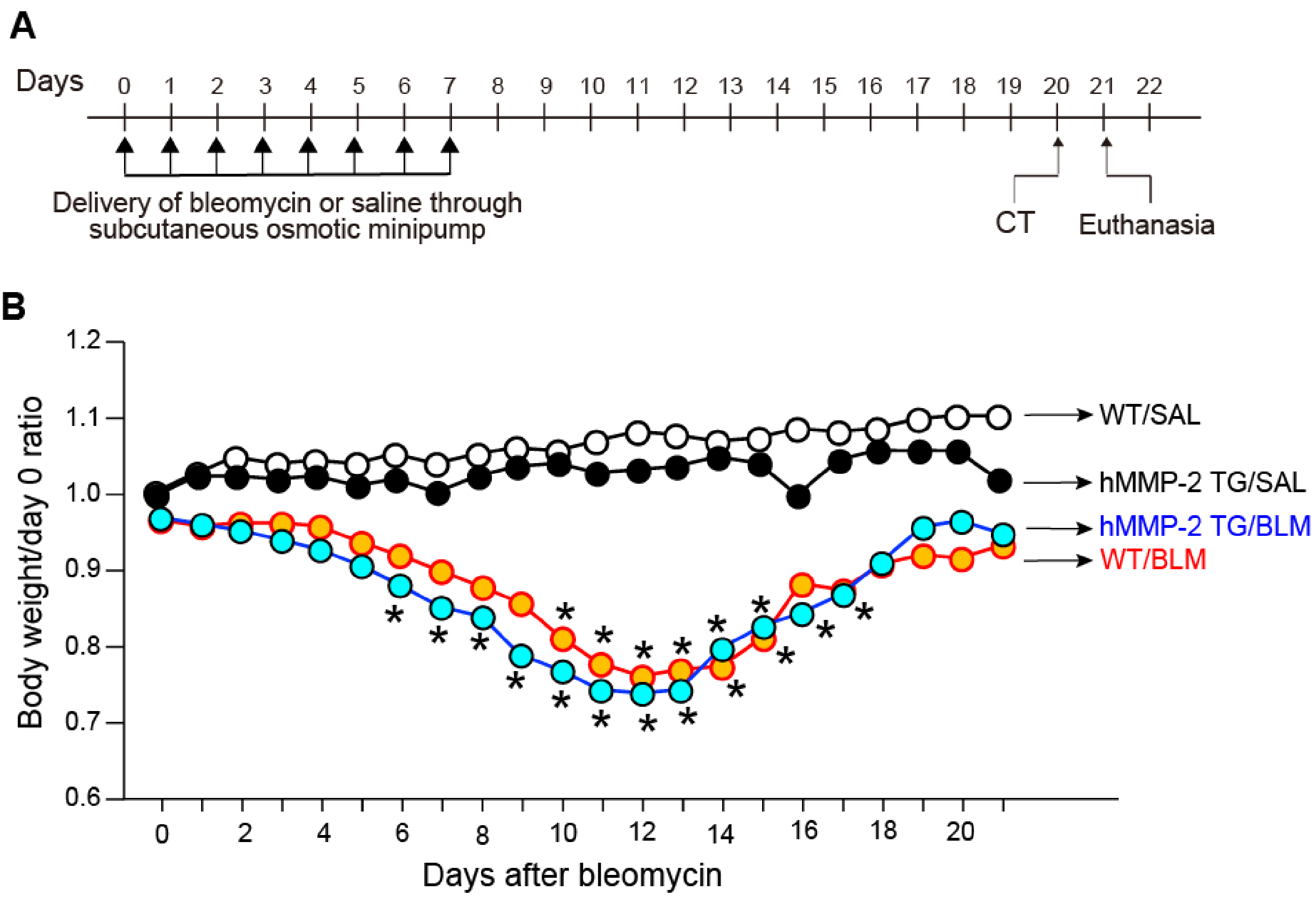

Supplementary Figure S1. No difference in body weight between WT and hMMP-2 TG mice with lung fibrosis. Mice received bleomycin or saline through osmotic minipumps for seven days. Lung fibrosis was evaluated on day 21 after the first day of bleomycin infusion (A). The body weight of the mice was measured daily and expressed as the ratio of body weight and body weight on day 0 (B). The number of mice: n=8 in WT/SAL, n=9 in hMMP-2 TG/SAL, n=16 in WT/BLM, and n=11 in hMMP-2 TG/BLM. All mice were female. Data are expressed as the mean. Statistical analysis by ANOVA with repeated measures and Newman-Keuls test. *$p<0.05$ vs. day 0.

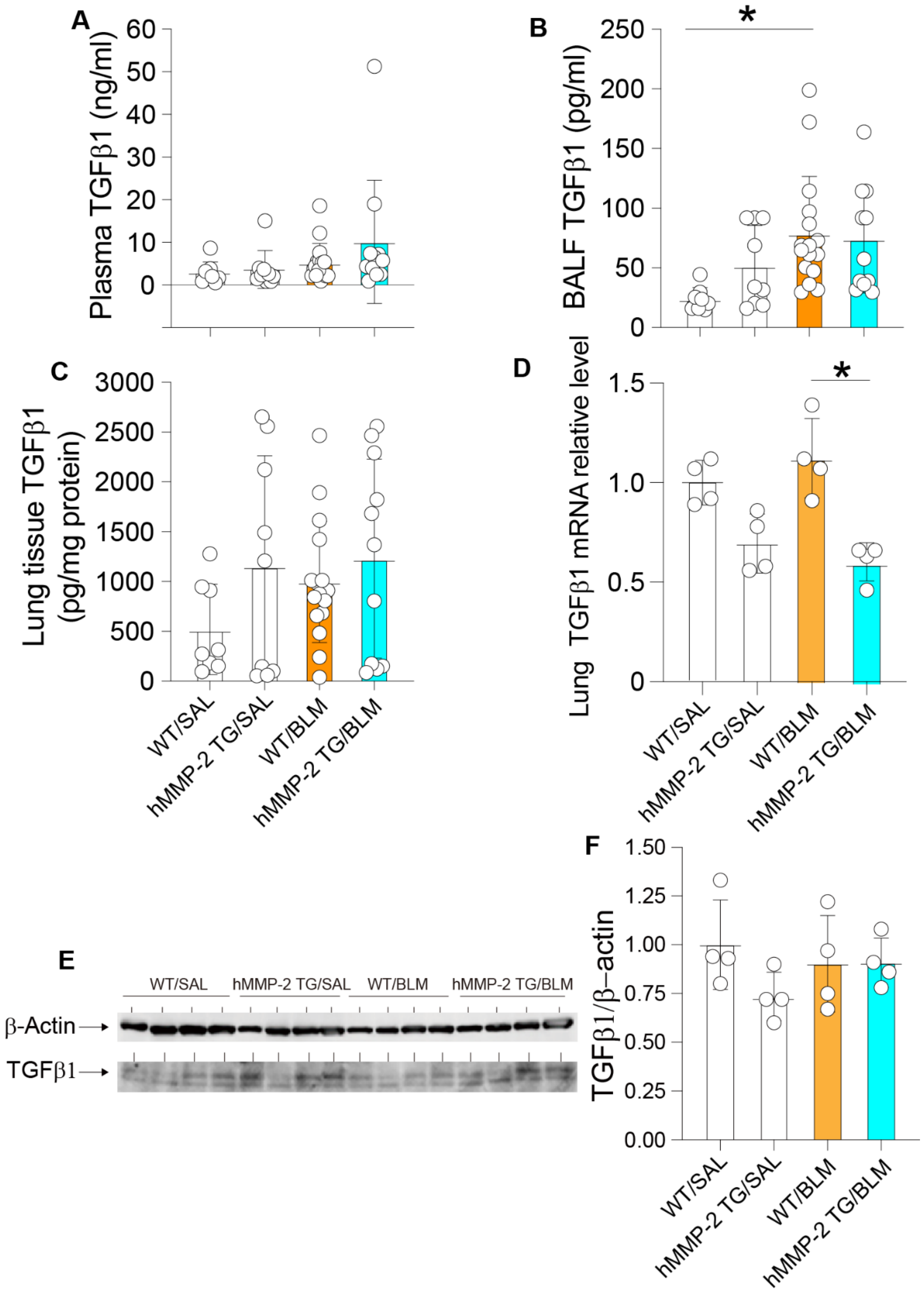

Supplementary Figure S2. No significant change in the levels of transforming growth factor-β1 level. A, B, C, the levels of transforming growth factor-β1 were measured by commercially available enzyme immunoassay kits following the manufacturer's instructions. The number of mice: n=8 in WT/SAL, n=9 in hMMP-2 TG/SAL, n=16 in WT/BLM, and n=11 in hMMP-2 TG/BLM. D, PCR was performed as described under materials and methods. n=4 in each group. E, F, Western blotting was performed using a commercially available antibody. n=4 in each group. Data are the mean ± S.D. Statistical analysis by ANOVA with Newman-Keuls test. *$p<0.05$. WT, wild-type; hMMP-2, human matrix metalloproteinase-2; SAL, saline; BLM, bleomycin.

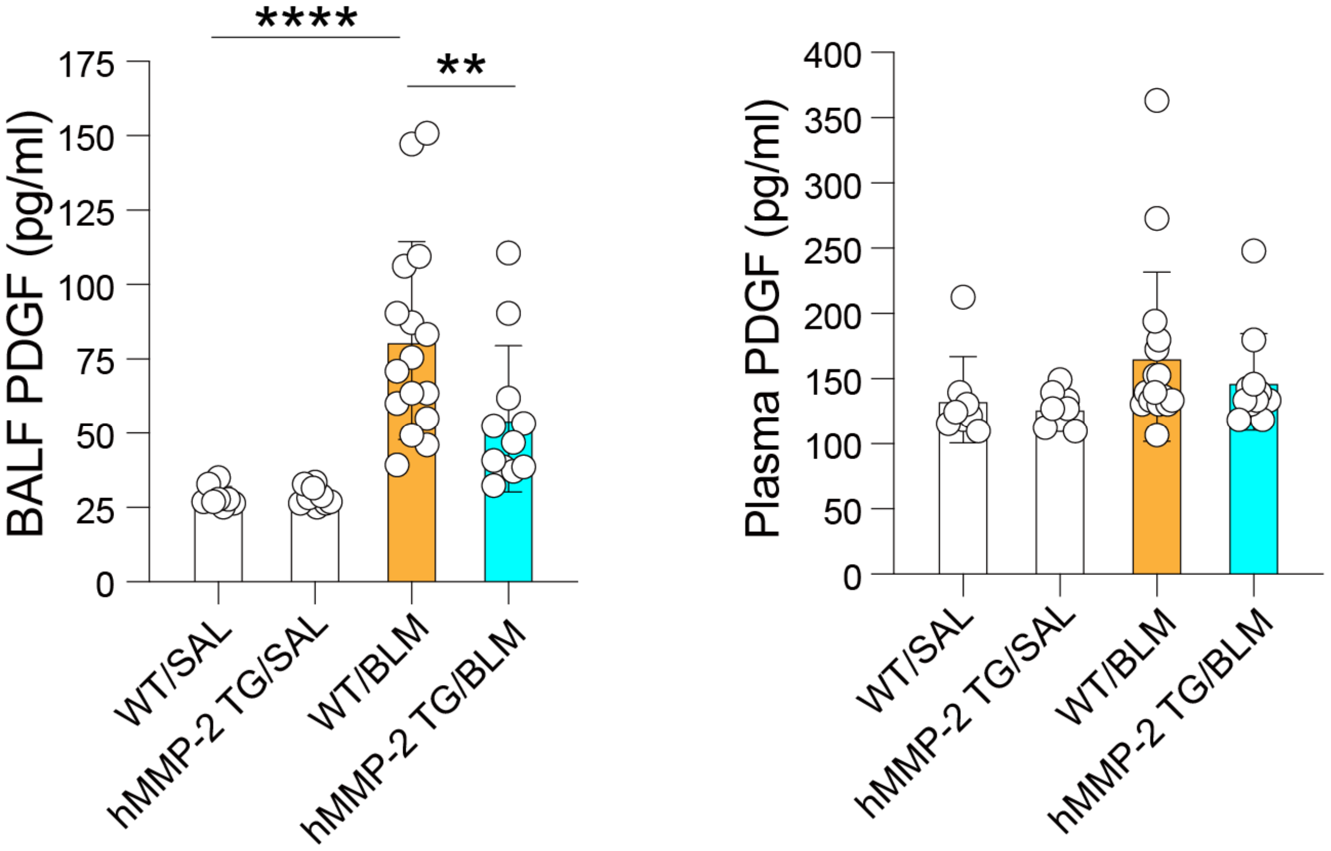

Supplementary Figure S3. Reduced platelet-derived growth factor in hMMP-2 TG mice. The platelet-derived growth factor (PDGF) level was measured as described under materials and methods. The number of mice: n=8 in WT/SAL, n=9 in hMMP-2 TG/SAL, n=16 in WT/BLM, and n=11 in hMMP-2 TG/BLM. Data are the mean ± S.D. Statistical analysis by ANOVA with Newman-Keuls test. ****$p<0.0001$; **$p<0.01$. WT, wild-type; hMMP-2, human matrix metalloproteinase-2; SAL, saline; BLM, bleomycin.

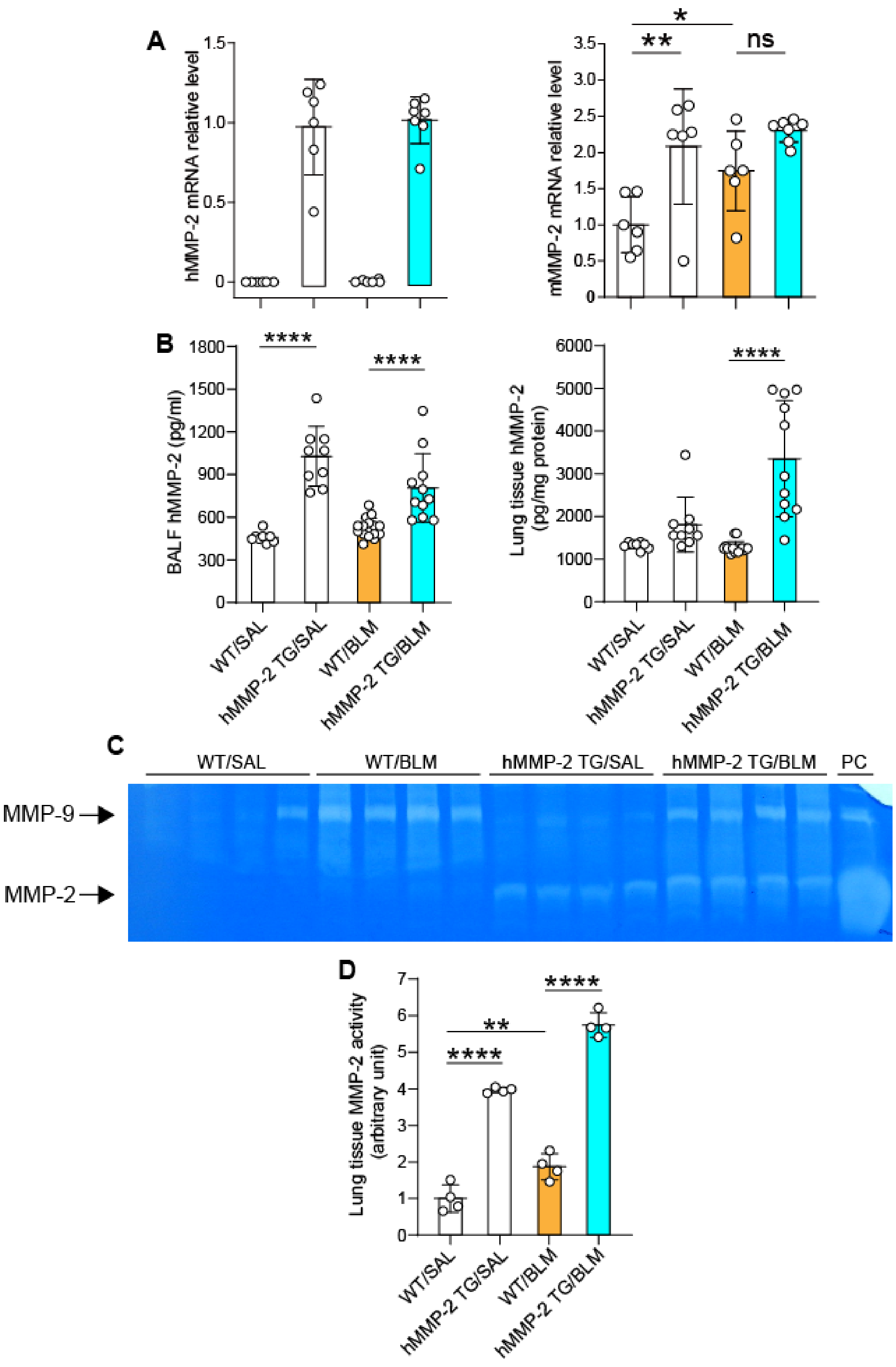

Supplementary Figure S4. Increased level and activity of MMP-2 in hMMP-2 TG mice. The levels of human MMP-2 were measured by polymerase chain reaction (A) and by commercially available enzyme immunoassay (B) and zymography (C, D) as described under materials and methods. The number of mice in A: n=6 in WT/SAL, hMMP-2 TG/SAL, WT/BLM, and n=7 in hMMP-2 TG/BLM. The number of mice in B: n=8 in WT/SAL, n=9 in hMMP-2 TG/SAL, n=9 in WT/BLM, and n=11 in hMMP-2 TG/BLM. The number of mice in C: n=4 in all groups. Data are the mean ± S.D. Statistical analysis by ANOVA with Newman-Keuls test. *p<0.05; **p<0.01; ****p<0.0001. WT, wild-type; hMMP-2, human matrix metalloproteinase-2; SAL, saline; BLM, bleomycin.

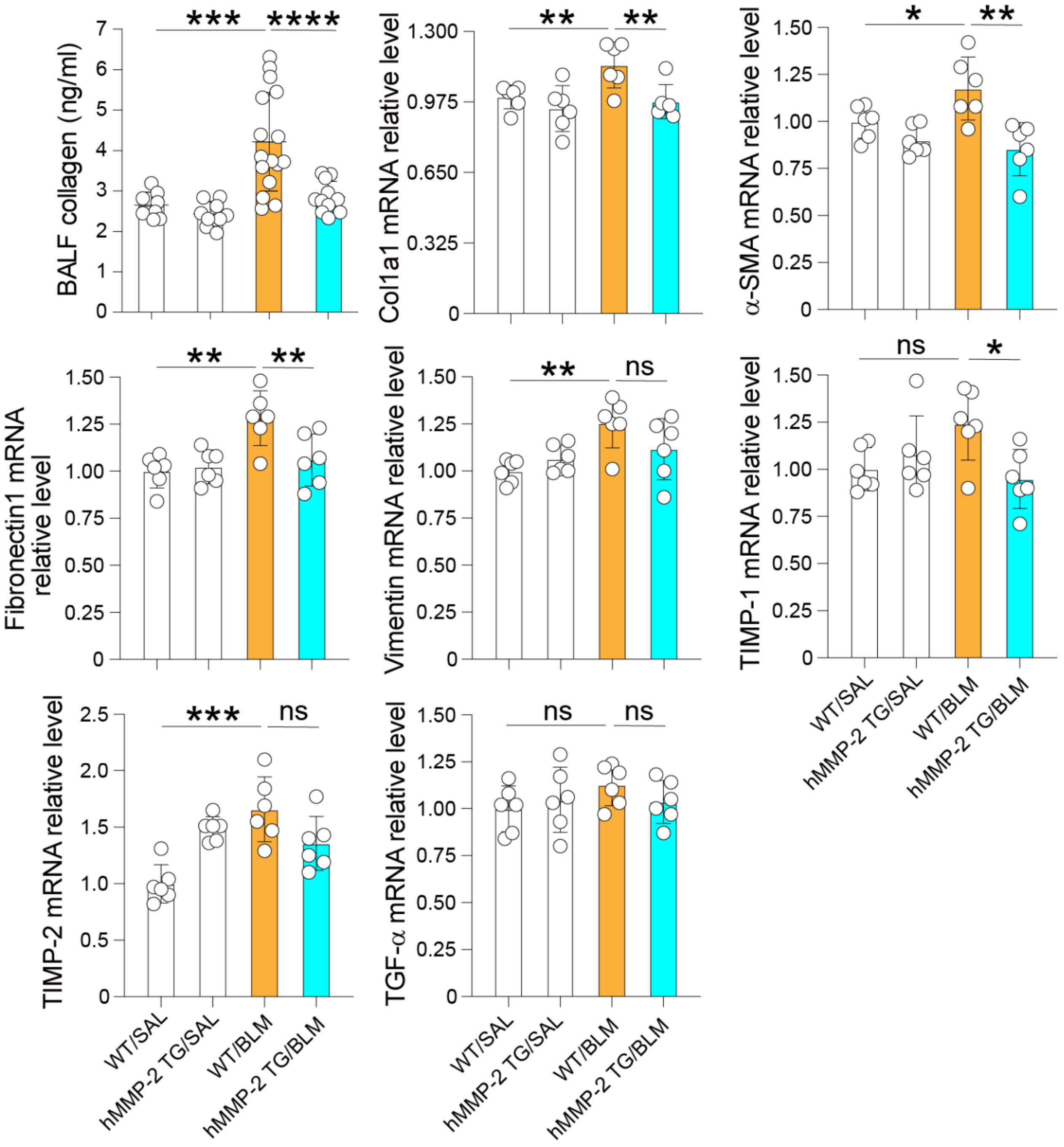

Supplementary Figure S5. The bronchoalveolar lavage fluid level of collagen, the mRNA expression of matrix components, inhibitors of metalloproteinases, and transforming growth factor-*α*. The bronchoalveolar lavage fluid (BALF) level of collagen was measured by immunoassay as described under materials and methods. The mRNA expression of matrix components and inhibitors of metalloproteinases was evaluated by RT-PCR as described under materials and methods. The number of mice was n=4 in all groups. Data are the mean ± S.D. Statistical analysis by ANOVA with Newman-Keuls test. *p<0.05; **p<0.01; ***p<0.001; ****p<0.0001. WT, wild-type; hMMP-2, human matrix metalloproteinase-2; SAL, saline; BLM, bleomycin.

	BALF all cells	BALF total macrophages	BALF total lymphocytes	BALF total neutrophils	BALF total protein	BALF IL-13	BALF MCP-1	BALF IFNγ	BALF IL-6	BALF IL-1β	BALF MCP-3	BALF CTGF	BALF TGFβ1	BALF collagen	BALF osteopontin	BALF hMMP-2	CT score	Ashcroft score	Collagen depositioon	Lung hydroxyproline
BALF all cells	1.0																			
BALF total macrophages	0.8*	1.0																		
BALF total lymphocytes	0.8*	0.8*	1.0																	
BALF total neutrophils	0.4	0.5	0.4	1.0																
BALF total protein	0.6*	0.7	0.9*	0.4	1.0															
BALF IL-13	0.4*	0.4*	0.5*	0.3	0.4*	1.0														
BALF MCP-1	0.7*	0.7*	0.9*	0.4	0.9*	0.5*	1.0													
BALF IFNγ	0.7*	0.7*	0.9*	0.4	0.7*	0.5*	0.9*	1.0												
BALF IL-6	0.5*	0.7*	0.8*	0.4	0.7*	0.4*	0.7*	0.8*	1.0											
BALF IL-1β	0.6*	0.6*	0.7*	0.4	0.6*	0.4*	0.7*	0.7*	0.6*	1.0										
BALF MCP-3	0.6*	0.5*	0.7*	0.4	0.6*	0.5*	0.7*	0.7*	0.4*	0.5*	1.0									
BALF CTGF	0.7*	0.5*	0.5*	0.4	0.4*	0.3*	0.4*	0.5*	0.4*	0.3*	0.3*	1.0								
BALF TGFβ1	0.4*	0.6*	0.6*	0.3	0.5*	0.6*	0.6*	0.6*	0.5*	0.4*	0.3*	0.1	1.0							
BALF collagen	0.7*	0.6*	0.7*	0.4	0.6*	0.3	0.7*	0.7*	0.6*	0.6*	0.4*	0.6*	0.3	1.0						
BALF osteopontin	0.6*	0.6*	0.8*	0.5	0.7*	0.5*	0.8*	0.7*	0.7*	0.5*	0.6*	0.4*	0.4*	0.6*	1.0					
BALF hMMP-2	-0.3*	-0.2	-0.2	-0.3	-0.1	0.1	-0.2	-0.2	-0.2	-0.3	-0.0	-0.4*	0.1	-0.4*	-0.0	1.0				
CT score	0.7*	0.5*	0.8*	0.5*	0.7*	0.4*	0.7*	0.7*	0.4*	0.6*	0.8*	0.5*	0.1	0.6*	0.7*	-0.5*	1.0			
Ashcroft score	0.7*	0.7*	0.9*	0.4	0.8*	0.5*	0.8*	0.8*	0.8*	0.7*	0.6*	0.5*	0.4	0.6*	0.8*	-0.3	0.8*	1.0		
Collagen deposition	0.6*	0.5*	0.5*	0.4	0.4*	0.2	0.4*	0.4*	0.3*	0.5*	0.4*	0.4*	0.2	0.5*	0.4*	0.3*	0.5*	0.5*	1.0	
Lung hydroxyproline	0.4*	0.5*	0.6*	0.4	0.6*	0.1	0.6*	0.6*	0.6*	0.3*	0.1	0.5*	0.2	0.3*	0.6*	-0.3*	0.5*	0.5*	0.5*	1.0

Supplementary Figure S6. Correlations between several parameters. The strength of the correlation between parameters was calculated using the Spearman correlation. BALF, bronchoalveolar lavage fluid; IL, interleukin; MCP-1, monocyte chemoattractant protein-1; MCP-3, monocyte chemoattractant protein-3; IFNγ, interferon-γ; CTGF, connective tissue growth factor; TGFβ1, transforming growth factor-β1; hMMP-2, human matrix metalloproteinase-2. *$p<0.05$.

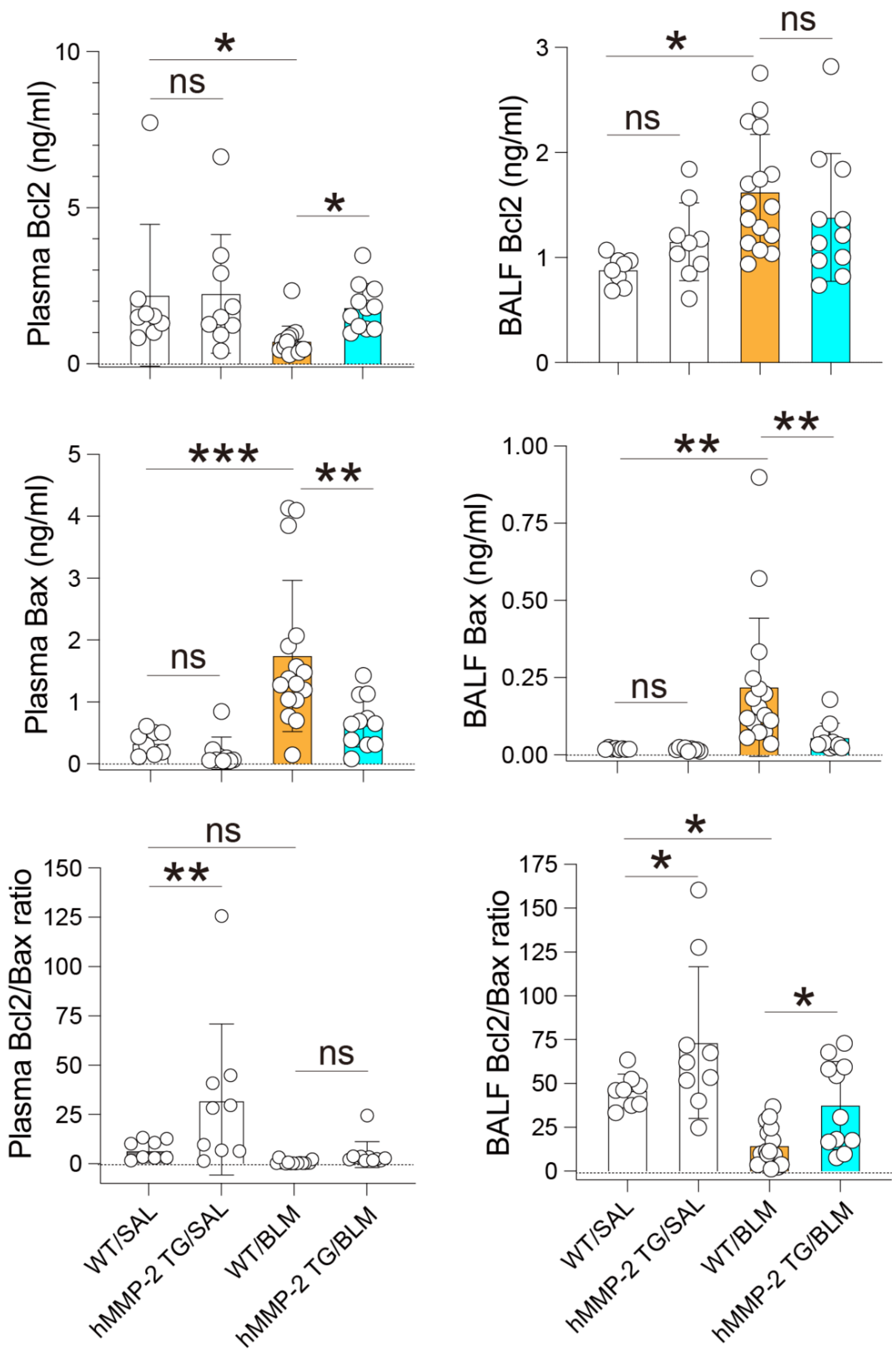

Supplementary Figure S7. Increased antiapoptotic protein level and decreased proapoptotic protein level in hMMP-2 TG mice. The plasma and bronchoalveolar lavage fluid (BALF) levels of Bcl2 and Bax were measured by commercially available enzyme immunoassay as described under materials and methods. The number of mice: n=8 in WT/SAL, n=9 in hMMP-2 TG/SAL, n=16 in WT/BLM, and n=11 in hMMP-2 TG/BLM. Data are the mean ± S.D. Statistical analysis by ANOVA with Newman-Keuls test. *p<0.05; **p<0.01; ***p<0.001. WT, wild-type; hMMP-2, human matrix metalloproteinase-2; SAL, saline; BLM, bleomycin.

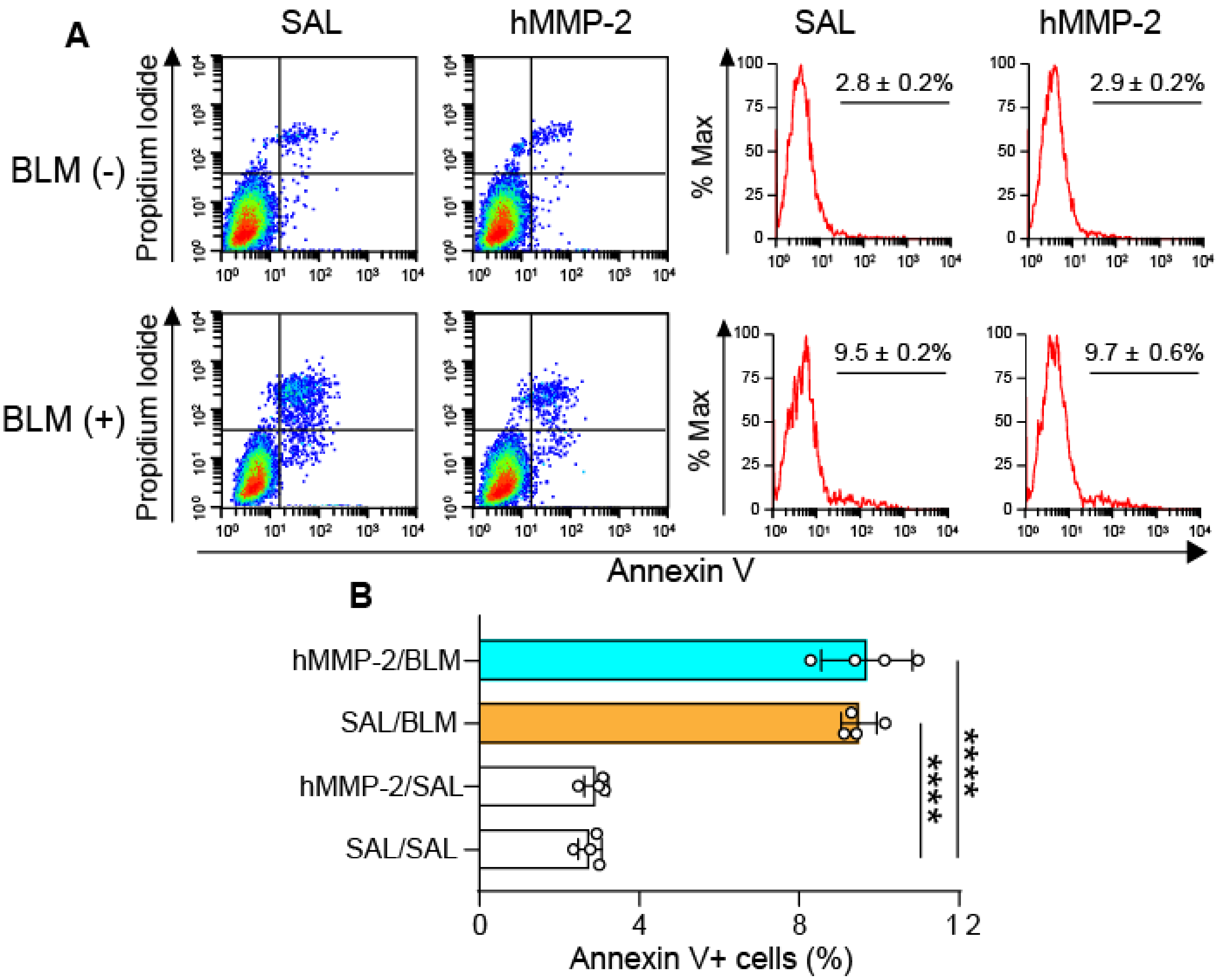

Supplementary Figure S8. Active hMMP2 does not inhibit apoptosis of lung fibroblasts. Murine lung fibroblasts were cultured and pretreated with active hMMP-2 (0.5 μg/ml) before stimulating with BLM (50 mU/ml) to evaluate apoptosis by flow cytometry. n=4 in each treatment group (A, B). Data are the mean ± S.D. Statistical analysis by ANOVA with Newman-Keuls test. ****$p<0.0001$; hMMP-2, human matrix metalloproteinase-2; SAL, saline; BLM, bleomycin.

Supplementary Table S2. Primers for RT-PCR

	Sequence (5' -> 3')	Tm	Reference	Location	Product size
mBIRC1a (NAIP1)					
Sense	TGCCCAGTATATCCAAGGCTAT	60.2	NM_008670	708-729	116 bp
Antisense	AGACGCTGTCGTTGCAGTAAG	62.6		823-803	
mBIRC1b (NAIP2)					
Sense	AGCTTGGTGTCTGTTCTCTGT	61.0	NM_001126182	1204-1224	180 bp
Antisense	GCGGAAAGTAGCTTTGGTGTAG	61.2		1383-1362	
mBIRC2 (c-IAP1)					
Sense	TGTGGCCTGATGTTGGATAAC	60.0	NM_007465	256-276	164 bp
Antisense	GGTGACGAATGTGCAAATCTACT	60.9		419-397	
mBIRC3 (c-IAP2)					
Sense	ACGCAGCAATCGTGCATTTTG	62.9	NM_007464	1073-1093	181 bp
Antisense	CCTATAACGAGGTCACTGACGG	61.6		1253-1232	
mBIRC4 (XIAP)					
Sense	CGAGCTGGGTTTCTTTATACCG	60.7	NM_009688	145-166	126 bp
Antisense	GCAATTT GGGGATATTCTCCTGT	60.4		270-248	
mBIRC5 (Survivin)					
Sense	GAGGCTGGCTTCATCCACTG	62.6	NM_009689	118-137	250 bp
Antisense	CTTTTTGCTTGTTGTTGGTCTCC	60.7		367-345	
mBIRC6 (Apollon)					
Sense	ACAGATTGTCTTACCTCTTGCCC	61.9	NM_007566	695-717	120 bp
Antisense	GCCACGAAGTGAAGGTCTCC	62.5		814-795	
mBIRC7 (ml-IAP)					
Sense	AGCCTCCTTCTACGACTGG	60.1	NM_001163247	291-309	245 bp
Antisense	GCAAAGGGGTGTAGGTCTGG	62.2		535-516	
mTGF-α					
Sense	CACTCTGGGTACGTGGGTG	61.7	NM_031199	217-235	136 bp
Antisense	CACAGGTGATAATGAGGACAGC	60.4		352-331	
mαSMA					
Sense	CAGGATGCAGAAGGAGATCAC	63.7	NM007392.2	1009-1029	364 bp
Antisense	TGTTGCTAGGCCAGGGCTAC	66.3		1372-1353	
mVimentin					
Sense	CGGCTGCGAGAGAAATTGC	61.8	NM_011701	550-568	124 bp
Antisense	CCACTTTCCGTTCAAGGTCAAG	61.3		673-652	
mMMP2					
Sense	CACCACCGAGGACTATGACC	61.4	NM_008610	954-973	122 bp
Antisense	TGTTGCCCAGGAAAGTGAAG	62.2		1075-1056	
hMMP2					
Sense	TACTGGATCTACTCAGCCAGCAC	61.8	NM_004530	1618-1640	184 bp
Antisense	CAGGATCCATTTTCTTCTTCACC	62.2		1801-1779	
mTIMP1					
Sense	TGTCCACAAGTCCCAGAACC	65.0	NM_011593	402-421	110 bp
Antisense	TCAGATTATGCCAGGGAACC	63.7		511-492	
mTIMP2					
Sense	TGGACGTTGGAGGAAAGAAG	64.1	NM_011594	305-324	97 bp
Antisense	TCTGGGTGATGCTAAGCGTG	65.1		401-382	
mFibronectin					
Sense	TTCAAGTGTGATCCCCATGAAG	60.0	NM_010233	7126-7147	154 bp
Antisense	CAGGTCTACGGCAGTTGTCA	61.5		7279-7260	
mGAPDH					
Sense	TGGCCTTCCGTGTTCCTAC	61.3	NM_008084	686-704	178 bp
Antisense	GAGTTGCTGTTGAAGTCGCA	60.9		863-844	
mCol1a1					
Sense	TAAGGGTCCCCAATGGTGAGA	67.4	NM007742	107-127	203 bp
Antisense	GGGTCCCTCGACTCCTACAT	64.2		309-290	
mBcl2					
Sense	AGCTGCACCTGACGCCCTT	69.6	NM_177410	344-362	192 bp
Antisense	GTTCAGGTACTCAGTCATCCAC	60.1		535-516	

mBax					
Sense	CGGCGAATTGGAGATGAACTG	68.7	NM_007527	190-210	161bp
Antisense	GCAAAGTAGAAGAGGGCAACC	63.8		350-330	
mBclxL					
Sense	AGGTTCCTAAGCTTCGCAATTC	64.4	NM_001289739	128-149	248bp
Antisense	TGTTTAGCGATTCTCTTCCAGG	64.2		375-354	
mMMP-9					
Sense	CCAGTATCTGTATGGTCGTG	64.2	NM_013599	1336-1355	106bp
Antisense	AGGTGCAGTGGGACACATAG	62.6		1441-1422	
mTNFα					
Sense	ACGTGGAACTGGCAGAAGAG	60.4	NM_013693	193-212	284bp
Antisense	CTCCTCCACTTGGTGGTTTG	60.5		476-457	
mIL-6					
Sense	CTGGTCTTCTGGAGTACCATAG	59.3	NM_031168	422-443	374bp
Antisense	AAGTCAGATACCTGACAACAGG	59.7		795-774	

BIRC1a,1b, 2, 3, 4, 5, 6, or 7: baculoviral iap repeat-containing 1a, 1b, 2, 3, 4, 5, 6, or 7. NAIP1, or 2: neuronal apoptosis inhibitory protein1, or 2. c-IAP1, or 2: cellular inhibitor of apoptosis protein 1, or 2. x-IAP: x-linked inhibitor of apoptosis protein. ml-IAP: melanoma inhibitor of apoptosis. TGF-β1: transforming growth factor-β1. GAPDH: glyceraldehyde 3-phosphate dehydrogenase. Bcl2: B-cell lymphoma 2. Bax: Bcl-2-associated X protein. BclxL: B-cell lymphoma-extra large. m, mouse; TIMP, tissue inhibitor of metalloproteinase; TGF-α, transforming growth factor-α; αSMA, α-smooth muscle actin; mTNFα, tumor necrosis factorα.
